# Toxic Alcohol Ingestion/Methanol Ingestion

**DOI:** 10.15766/mep_2374-8265.10740

**Published:** 2018-08-17

**Authors:** Mary Wittler, Mary Claire O'Brien, David A. Masneri

**Affiliations:** 1 Assistant Professor, Department of Emergency Medicine, Wake Forest Baptist Medical Center; 2 Professor, Department of Emergency Medicine, Wake Forest Baptist Medical Center; 3 Emergency Medicine Simulation Director, Wake Forest Baptist Medical Center

**Keywords:** Toxic Alcohol Ingestion, Methanol Intoxication

## Abstract

**Introduction:**

Methanol poisoning is an uncommon life-threatening ingestion associated with significant morbidity and requires prompt diagnosis and management for the best possible outcome. We created a simulation case that challenges learners to analyze case information, construct a differential diagnosis of an anion gap metabolic acidosis, narrow the differential based on reasoning, and empirically initiate management.

**Methods:**

The simulation case was designed for emergency medicine residents and pediatric emergency medicine fellows. The activity began with a brief overview of the monitors, equipment, and simulation experience. First-year residents managed the case as a team of two. Second- and third-year residents and fellows managed the case alone. The learners had 15 minutes to complete a focused history and physical exam, request and interpret labs and studies, provide stabilization of life threats, and initiate specific interventions based on a presumptive diagnosis. The simulation was followed by a 20-minute facilitated debrief session that reviewed key learning points and learner performance based on an evaluation checklist.

**Results:**

Residents completed a six-question, 5-point Likert-scale postparticipation questionnaire. Overall, residents reported a high degree of satisfaction with the simulation experience. The case and debrief were effective in meeting the educational objectives and proved to be an effective modality to fill this educational gap.

**Discussion:**

This simulation experience successfully exposed residents to the uncommon presentation of methanol poisoning. The simulation experience effectively closed the identified educational gap and provided an experiential learning opportunity that accomplished the targeted learning objectives.

## Educational Objectives

By the end of this session, learners will be able to:

1. Identify the signs and symptoms of toxic alcohol/methanol ingestion in a simulated case.
2. Discriminate the causes of anion gap metabolic acidosis.
3. Describe the metabolism of methanol and the pathophysiology as it relates to clinical presentation.
4. Explain the utility for sodium bicarbonate administration in this poisoning.
5. Differentiate the treatment goals for this poisoning and define the specific indications for treatment interventions.
6. Explain the utility of hyperventilation to prevent worsening acidemia postintubation.

## Introduction

Methanol poisoning is a life-threatening ingestion associated with significant morbidity and mortality. Seizures, cerebral edema leading to herniation, and cerebral infarcts are often preterminal events. Mortality is more likely to occur in patients who present with coma and severe metabolic acidosis.^1–3^ In survivors, sustained visual sequelae and a Parkinsonian-like syndrome are possible.^4,5^ Based on the 2016 annual report of the American Association of Poison Control Centers' National Poison Data System, methanol accounted for 1,987 of the approximately two million reported human exposures.^6^ The majority of methanol exposures were unintentional. Major clinical outcomes and deaths were reported for 20 (1%) and 16 (<1%) cases, respectively.^6^ Immediate recognition and timely management interventions can be lifesaving. Emergency medicine (EM) residents need to have familiarity with this ingestion in order to recognize the case presentation and initiate management interventions. Despite this need, many residents at our institution graduate without ever seeing a single case. In toxic alcohol ingestion cases, the initiation of lifesaving interventions can be significantly delayed secondary to diagnostic uncertainty and/or desire to have a confirmatory test prior to definitive management (primary author's observation). These challenges led to the development of a methanol poisoning simulation case to use for educational purposes with our EM residents.

A simulation exercise was adapted from a real case to provide experiential learning to EM residents and pediatric EM fellows, with the goals being to allow learners to analyze case information, construct a differential diagnosis of an anion gap metabolic acidosis (AGMA), narrow the differential based on reasoning, and empirically initiate the multimodal management interventions for this poisoning. The case management entails initiation of life-stabilizing interventions and the application of multiple management strategies to provide care. This simulation case requires residents to deliberate alternative diagnoses and empirically initiate specific treatments; this process reflects the real-life clinical decision-making process that occurs in the emergency department. A search of *MedEdPORTAL* revealed a previously published toxic alcohol (ethylene glycol) simulation case.^7^ However, to date, no case specific to methanol toxicity has been found.

## Methods

### Development

At Wake Forest Baptist Medical Center, the EM residents (PGY 1-PGY 3) participate in a three-armed simulation curriculum consisting of High-Fidelity Pediatric Simulation, High-Fidelity Joint Trauma Simulation, and in situ Emergency Department Low-Fidelity Curriculum. This case was presented in the Emergency Department Low-Fidelity Curriculum. The simulation case exercise (Appendix A) was rotated through this required curriculum on a repeat interval spaced across 3 years. While learners needed no specific prerequisite preparation, most had exposure to analyzing an AGMA as well as basic knowledge for stabilization of life-threatening illnesses. The facilitators included a board-certified medical toxicologist and the resident simulation director. Across 3 years, 30 PGY 1-PGY 3 residents and four pediatric EM fellows participated in this simulation case.

### Equipment/Environment

If a PGY 1 was the targeted learner, he/she was teamed with a second resident to run the case. If a PGY 2 or PGY 3 was the targeted learner, he/she ran the case, and a second resident acted to provide clinical interventions or “phone a friend” as needed. The simulation was performed in the emergency department. Simulation equipment included a simple mannequin, a programmable monitor to display vital signs, heart monitor leads, blood pressure cuff, and pulse oximeter. Supplemental oxygen by nasal cannula and face mask was available. Standard resuscitative equipment, defibrillator/pacemaker, and intubation equipment were present. All case information is included in the simulation case file (Appendix A). All case diagnostic information, including X-ray images, EKG, and laboratory values, is listed in the supplemental case findings document (Appendix B).

### Personnel

As implemented at our institution, the simulation session required two facilitators. One faculty member provided case information, and one simulation faculty updated vital signs and tracked interventions and requests for further information. The faculty member providing case information played the role of emergency medical services, family, and all consultants. The second facilitator helped to organize requested equipment, deliver medications and fluids, update vital signs, and track requests for labs and verbalized interventions. Alternatively, a nurse could be embedded into the case to increase realism.

### Implementation

Over a period of 3 years, we ran this simulation exercise with 30 PGY 1-PGY 3 EM residents and four pediatric EM fellows. No changes were made to the original case. Cues for the facilitator were added during this time based on resident management of the case.

At the start of the simulation session, we briefed each team about the simulation environment and provided instructions for the simulation activity. The briefing included instruction to verbalize every request (place an IV, draw blood, etc.) and provided a general overview of case flow. We informed the residents of the 15-minute time limit to perform the components of the case and told them to include a focused history and physical exam, order and interpret studies, make treatment plans, secure disposition, and discuss the case with consultants. The residents were allowed to navigate through the case without interruption from facilitators. Requested studies (EKG and chest X-ray) were provided when requested. Laboratory results were provided at the facilitator's discretion for best timing of delivery; they were not provided before a complete history and physical exam had been performed. Definite testing for toxic alcohols was not available during the simulation, and treatment decisions were made based on empiric evidence. The following studies and labs were available if requested: EKG, chest X-ray, CT head, comprehensive metabolic panel, complete blood count, arterial blood gases, urinalysis, urine drug screens, urine pregnancy test, lactic acid level, serum toxicology levels (salicylate, acetaminophen, ethanol), serum osmolality level, and CO-oximetry levels.

We used a flip chart to track treatment interventions and requests for laboratory values. The main facilitator was available for phone consultation with toxicology. For senior residents, the toxicologist confirmed antidote suggestions verbalized by the resident, gave specific dosing information, and provided further treatment recommendations. For junior residents, the toxicologist was more helpful in initiating antidote management decisions. The facilitator also role-played as an intensivist and nephrologist for telephone consult. If either was called prior to verbalization of diagnosis and initiation of management interventions, then he/she was unavailable until diagnosis of suspected toxic alcohol ingestion had been made.

### Assessment

At the end of the learning experience, the learners voluntarily completed an anonymous, six-question questionnaire concerning the event (Appendix C). The questionnaire used a 5-point Likert scale. This questionnaire was developed from adaption of the Effective Evaluation: A Toolkit for Evaluating Presentations and Training publication.^8^

### Debriefing

We cofacilitated a debrief session at the end of each simulation scenario. We utilized a debriefing strategy similar to the PEARLS debriefing framework.^9^ The components of the debriefing were (1) learner self-assessment, (2) focused facilitation, and (3) directive feedback and teaching. We started the debrief by asking the primary learner to reflect on how he/she felt the scenario had gone. This elicited the learners' reactions and provided a “clear the air” moment that allowed the learners to raise their concerns and/or questions about the simulation and/or case management. This functioned as a springboard to explore the learners' views, to legitimize or correct the learners' thought processes, and to align the teaching objectives with both learner and educator concerns. An advocacy-inquiry approach, aimed at understanding the thought processes behind the actions taken, was helpful in guiding this discussion. All learners were asked to provide observations regarding care decisions and to reflect upon the drivers behind their actions. We then offered directive feedback and teaching that represented a combination of witnessed actions or decisions made by the resident during the simulation and included a review of the critical actions checklist (Appendix D). We incorporated a didactic review that covered the teaching points into the debrief session (Appendix E). These topics were reviewed at the whiteboard, highlighting major teaching points related to the case.

## Results

Since initiation of this case simulation, it has been successfully used with 30 EM PGY 1-PGY 3 residents and four pediatric EM fellows. As depicted in the Table, the questionnaire results showed a favorable reaction to the educational experience for all questions answered. The majority of respondents felt that the event was effective in improving their recognition of the signs and symptoms of methanol toxicity. Most learners found that the case was helpful in promoting critical evaluation of an AGMA. The majority of learners reported increased familiarity with treatment modalities and the indications to initiate treatment options. Additionally, the didactic review of the metabolism of methanol and the pathophysiology as it relates to clinical presentation contributed to meeting our educational objectives.

**Table.** Toxic Alcohol/Methanol Questionnaire Responses (*n* = 30)

Statement	Strongly Disagree	Disagree	Neutral	Agree	Strongly Agree
Overall, I am satisfied with this educational event.	0%	0%	4%	37%	59%
I am better equipped to identify the signs and symptoms of a toxic alcohol/methanol intoxication.	0%	0%	9%	43%	48%
I am more confident in my ability to critically evaluate causes of acid-base disturbances.	0%	0%	16%	43%	41%
I have better understanding of the treatment goals for this poisoning and specific indications for management options.	0%	0%	8%	41%	51%
This event enhanced my knowledge of methanol toxicity.	0%	0%	2%	24%	74%
The debrief was effective in presenting the educational objectives.	0%	0%	8%	32%	60%

We did not specifically ask for comments in the questionnaire; however, unsolicited comments included the following:

• “Very useful simulation.”
• “This was great, I've never seen this type of ingestion.”
• “I can now describe the relationship of osmols to alcohols and AG to metabolites.”
• “I understand the topic better than before the simulation.”
• “I have improved understanding of how to use lab studies in suspected toxic alcohol ingestion to start management.”

## Discussion

This simulation exercise was successful in exposing residents to methanol poisoning, providing experiential learning during deliberate evaluation of case data and management decisions. All learners reported value for the educational experience, based on questionnaire data. The overwhelmingly positive reception, as supported by the questionnaire results, indicates that this simulation event is an appropriate modality to achieve the stated learning objectives. We believe this experience effectively closed the learning gap identified at our institution. It provided an experiential learning opportunity to challenge the learner's knowledge base and management decisions for methanol toxicity.

The case was adapted from a real case, and as such, the laboratory values and case information are realistic. This case was specifically designed for use with EM residents and pediatric EM fellows; however, it could be used by other learners, including pediatric and internal medicine residents and critical care fellows. We ran the simulation in the emergency department with an adult-size mannequin with a portable, programmable monitor to display vital signs. However, the case could be run in a simulation center. The case simulation was delivered by two faculty facilitators, including a medical toxicologist for case and content delivery and the EM simulation director to assist with interventions, adjust programming, and track requests for further lab studies and interventions. However, roles can vary depending on the number of participants in the simulation session and on facilitator availability. The simulation can be implemented using a single participant (or teams) and one facilitator/operator who provides oral feedback and plays additional historical and consultant roles. Using physical actors such as nurses or medic providers can enhance realism but is not required to successfully implement the case. The debrief materials feature slide content notes to facilitate delivery by faculty having less familiarity with the content; successful reproducibility should be obtainable. We used a single whiteboard to track interventions and requests for information during the case and to discuss the didactic learning objectives. However, other means (e.g., PowerPoint) of delivering teaching content are also an option.

The case was created to fill a void at our institution in exposure to this type of poisoning. Our institution does not have real-time testing of toxic alcohols, as is the case at most institutions. Even at institutions with in-house testing for toxic alcohols, levels take time to result. As such, this patient population should be managed empirically. Junior residents were more likely to fail to troubleshoot the AGMA by addition of other laboratory studies (lactic acid level, consideration of ketosis) as a means of narrowing the differential. Senior residents often hesitated to initiate treatment despite suspecting that the case was a toxic alcohol ingestion. In learner reflection, this was born out of a lack of previous experience managing this type of ingestion and/or a hesitation secondary to diagnostic uncertainty. A frequent learner comment was “I'm not sure how to proceed” or “I've never seen this before.” Such diagnostic uncertainty and lack of availability for confirmatory testing reflect clinical practice in most emergency departments. An additional management error was learner unpreparedness for the information provided by the Poison Control Center. As this type of intoxication requires multimodal management steps, specific and lengthy recommendations may include fomepizole dosing recommendations, initiation of co-factor therapy, initiation of bicarbonate bolus and drip, the need to consult nephrology for dialysis, and the need to send toxic alcohol levels.

The case content has been adapted for use as an oral board test case, for one-on-one teaching, and as a case example for problem-based learning discussion (PBLD). The case easily adapts to an oral board review case by simply pasting the labs and studies on single pages to provide to the learner when queried. Additionally, the critical actions checklist adapts from formative to summative feedback without changing the critical interventions. Otherwise, all case content needed to deliver the case is present in the case summary. For use in a PBLD, the case content can be delivered to the learners, utilizing the debrief materials to direct discussion about the metabolism, pathophysiology, presentation, and management interventions. In these environments, learners have included PGY 1-PGY 3 EM residents and pediatric EM fellows, and the case content has been delivered to more than 110 learners.

A limitation of this educational scholarship is that our evaluation tool measured only the learners' reaction to the simulation event and their perceptions of learning. Because of the timing of this simulation event, we did not administer pre- and posttests for knowledge gained. A pretest would have defeated the purpose of managing an unknown case and biased the learner to the educational goals. A posttest, if employed, would have identified continued knowledge gaps but would not have measured knowledge gained through the educational process. Finally, because of the infrequency of this presentation in the emergency department, it would be extremely difficult to measure a change of behavior or transfer of knowledge in the clinical setting by use of this simulation.

Overall, residents reported that the learning experience was beneficial and filled an educational gap identified in our department. This case has been incorporated into a practice case for oral board testing and is a representative case for use in the EM resident toxicology curriculum as a PBLD. As this is an uncommon ingestion, our hope is that the experiential learning process translates to prompt recognition and imitation of treatment in the emergency department to optimize patient outcome.

## Appendices

A. Methanol Simulation Case.docx
B. Methanol Supplemental Case Findings.ppt
C. Methanol Questionnaire.docx
D. Methanol Evaluation Form.doc
E. Methanol Case Debriefing.pptx All appendices are peer reviewed as integral parts of the Original Publication.
