# Supplementary material for: Toxic Alcohol Ingestion/Methanol Ingestion
Source: MedEdPORTAL. 2018 Aug 17;14:10740. doi: 10.15766/mep_2374-8265.10740 (PMC6342378; doi:10.15766/mep_2374-8265.10740)
Supplement: Supplementary file 1 — A. Methanol Simulation Case.docx B. Methanol Supplemental Case Findings.ppt C. Methanol Questionnaire.docx D. Methanol Evaluation Form.doc E. Methanol Case Debriefing.pptx [file mep-14-10740-s001.zip › A._Methanol_Simulation_Case.docx]

| **Appendix A: MedEdPORTAL Simulation Case**  **SIMULATION CASE TITLE:** Toxic Alcohol Ingestion/Methanol Ingestion  AUTHORS: Mary Wittler, MD., Mary Claire O’Brien, MD., David Masneri, DO | |
| --- | --- |
| **PATIENT NAME:** Ms. Anna Johnson  **PATIENT AGE:** 45 years old  **CHIEF COMPLAINT:** Altered Mental Status (AMS) | |
|  | |
| **Brief narrative description of case** | A 45 year-old-hospital employee didn’t show up to work or respond to telephone calls. After being unable to establish contact for 6 hours, concerned colleagues notified the police department, who subsequently found the patient unresponsive in bed at home. EMS transports the patient to the Emergency Department for further care.  On ED arrival, the patient has AMS with a GCS of 7 (E2V1M4) and is otherwise hemodynamically stable. The learner should check a glucose secondary to AMS, and prepare to intubate the patient. Depending on learner level, specific drug choice and dosing for rapid sequence intubation can be discussed, and successful intubation confirmed. Secondary evaluation of the patient reveals no other clues for the reason of AMS, and the learner must work through an AMS differential diagnosis and order appropriate clinical testing. Resulting labs show a profound Anion Gap Metabolic Acidosis (AGMA) that requires further consideration of possible etiologies. Initial resuscitative considerations include fluid resuscitation and administration of a bicarbonate bolus and drip. Definitive therapy should be initiated for suspected toxic alcohol ingestion, to include administration of Fomepizole, consultation with nephrology for dialysis, administration of co-factor therapy, and consultation with MICU for bed placement. The local Poison Control Center (PCC) is available for consultation. |
| **Primary Learning Objectives** | By the end of this session, learners will be able to:   1. Identify the signs and symptoms of toxic alcohol/methanol ingestion in a simulated case 2. Discriminate the causes of anion gap metabolic acidosis 3. Describe the metabolism of methanol and the pathophysiology as it relates to clinical presentation 4. Explain the utility for sodium bicarbonate administration in this poisoning 5. Differentiate the treatment goals for this poisoning and define the specific indications for treatment interventions 6. Explain the utility of hyperventilation to prevent worsening acidemia post intubation |
| **Critical Actions** | 1. Check blood sugar on an altered patient 2. Perform focused history and physical exam based presentation 3. Intubate patient secondary to altered mental status 4. Initiate normal saline fluid resuscitation 5. Troubleshoot differential of AGMA by sending lactic acid and salicylate levels 6. Start fomepizole 7. Discuss case with nephrology for hemodialysis and intensive care unit admission |
| **Learner Preparation** | Depending on the level of the learner and the focus of the educator, the learners may prepare for assessment of an acid-base disturbance before this simulation activity, and/or review the management of toxic alcohols. One preparatory assignment for the evaluation of acid-base disorders and the evaluation and management of toxic alcohols are listed below:  Kelen G, Nicolaou D, Cline D. Chapter 15: Acid-Base Disorders. In Tintinalli JE, Stapczynski JS, Ma OJ (eds) Tintinalli's Emergency Medicine: A Comprehensive Study Guide. 8^th^ ed. New York, NY: McGraw-Hill; 2016.  Brent J. Fomepizole for Ethylene Glycol and Methanol Poisoning. NEJM 2009; 360:2216-23. |

| Initial Presentation | | | |
| --- | --- | --- | --- |
| **Initial vital signs** | BP 128/72 mmHg, HR 102 beats/min, RR 24 breaths/min, Sats 100% (room air), Temp 98.4^o^ F (rectal)  Weight 60 Kg | | |
| **Overall Appearance** | Upon entry to the room, the learners will see a fully dressed patient who is lying in bed and appears to be unresponsive. The patient appears to be breathing fast, although unlabored. EMS transported the patient secondary to AMS. No monitors are on the patient. | | |
| **Actors and roles in the room at case start** | Roles can vary depending on the number of participants in the simulation session and facilitator availability. This case can be implemented using a single participant and one facilitator/operator who can provide oral feedback and play additional historical or consultant roles via voice only. A nurse or EMS personnel can be used at the bedside to help deliver care to the patient, including to perform medical interventions, as well as to provide ancillary support.  Only those interventions requested by the learner(s) should be performed. The main learner will act as the primary physician and do the primary evaluation of the patient to include obtaining a history, conducting a physical exam, and ordering any necessary medications or other interventions. The primary learner may perform any needed procedures, or may delegate these to other providers if multiple participants are present. Other learners in the scenario can serve as collaborators, assistants for any necessary procedures, or observers.  A faculty instructor is present, either in person, or in the control room for simulation equipment. This faculty instructor may serve as the voice of the EMS provider, as well as consultants including the poison control center, the nephrologist, and the intensivist. The faculty instructor may operate the simulation equipment by triggering manual changes as scripted, or allow this operation to be performed by an assistant. The faculty instructor observes the performance of the team, provides feedback, and facilitates the debriefing session.  EMS report (transitioning care): The patient is a hospital employee and didn’t show up to work for 1 day. The police department was notified, who found the patient unresponsive at home. On our arrival, the patient was found minimally responsive in bed. No empty medication bottles or other substances were found next to the patient. We didn’t see any evidence of trauma or anything else unusual when leaving the house. | | |
| **HPI** | At the start of the scenario, the EMS providers have the following information:   - Patient name: Ms. Anna Johnson - Weight: 60 Kg - Demographics: 45 y/o female - ED arrival information: Brought by EMS from home - Chief complaint: AMS - Significant history/details: The patient is a hospital employee who didn’t show up for work   Further historical information, including review of systems is unobtainable from the patient secondary to AMS.  The following Information can be found from chart review, if requested:   - Allergies: NKDA - Home medications: none - Medical history: none - Surgical history: none - Social history: no known use of ethanol, smoking, or drugs - Family comments: A brother, James Johnson, who has been informed by police that his sister was transported to the hospital, can be contacted to provide further information: - He is generally unfamiliar with her health history, and knows of no medical illnesses - She recently divorced after 20 years of marriage. - She recently expressed feelings of depression related to going thru the divorce and living alone. - She had no previous history of depression, suicide attempt, nor has she expressed suicidal ideations. - He knows of no history of drug or alcohol abuse. | | |
| **Past Medical/Surgical History** | **Medications** | **Allergies** | **Family History** |
| None | None | No known allergies | None contributory |
| **Physical Examination** | | | |
| **General** | Patient has a depressed level of consciousness. No evidence of trauma. She has mild tachypnea. | | |
| **HEENT** | Atraumatic and normocephalic. Pupils are 6mm, equal, and un-reactive to light bilaterally. Optic discs have hyperemia bilaterally. Conjunctiva is normal. Mucous membranes are moist. Oropharynx is clear with moist mucus membranes, but pooling secretions and absent gag reflex are noted. | | |
| **Neck** | No jugular venous distention; trachea midline. | | |
| **Lungs** | Mild tachypnea is present, otherwise clear to auscultation. | | |
| **Cardiovascular** | Regular rate and rhythm. No murmurs or rubs noted. | | |
| **Abdomen** | Normal bowel sounds. Non-distended bladder. No grimacing with palpation. | | |
| **Back** | Normal. | | |
| **Neurological** | No response to verbal stimuli. Withdraws to pain (M4) and opens eyes briefly to pain (E2). No speech to stimulation (V1) (GCS 7 - E2V1M4). Corneal reflex is intact, but no gag reflex is present. | | |
| **Skin** | Warm and dry. No diaphoresis or rashes noted. | | |
| **GU** | Guaiac negative stool. | | |
| **Extremities** | No cyanosis, clubbing, edema. | | |

| Instructor Notes - Changes and CASE Branch Points | | |
| --- | --- | --- |
| Intervention / Time point | Change in Case | Additional Information |
| IV saline may be administered at any time point | None | Nurse may prompt “I’ve placed an IV, do you want me to administer fluids?” |
| Check blood sugar secondary to AMS  *Scenario time = initial assessment* | Bedside accucheck = 90 | Nurse may prompt “Do you want me to check anything now since the patient is altered?” |
| Intubate patient secondary to GCS 7, no gag reflex, pooling secretions  *Scenario time = 1-2 minutes (initial stabilization)* | ETT visualized passing cords | Nurse may prompt, “Do you want me to call respiratory to help manage the patient’s airway?” |
| Confirm placement of ETT  *Scenario time = immediately after intubation* | Successful intubation, as determined by auscultation, end tidal CO2 monitor, and CXR results (available in Appendix B) | Nurse may prompt “Do you want me to call the X-ray technician?” |
| ETT placement is low  Scenario time = visualization of post-intubation CXR | When recognized and corrected by learner, facilitator announces “you’re ETT is now in good position” | Facilitator may prompt “what do you think of the ETT placement?” |
| Post-intubation VS  *Scenario time = immediately after intubation* | BP 112/65 mmHg, HR 110, RR 14 breaths/min, Sats 100% |  |
| Standard lab and tests should be ordered to evaluate patient’s AMS  *Scenario time = 4 minutes* | None | Nurse may prompt “What blood work do you want me to send?” or “Do you want any other imaging?” |
| Lab tests arrive  *Scenario time = 6 minutes* | Additional lab test may be needed to further evaluate AGMA (aspirin level, lactic acid, urine and/or serum ketones) | Learners should interpret/troubleshoot initial laboratory studies with AGMA. Facilitator may prompt “What is your differential diagnosis for AGMA?” |
| Treatment with IV sodium bicarbonate  *Scenario time = 8 minutes* | None | RT can prompt “her pre-intubation and post-intubation blood gases have resulted, that’s the worst acidemia I’ve seen” |
| Treatment with fomepizole  *Scenario time = 10 minutes* | All laboratory studies related to AGMA differential are available | Poison Center is available to discuss empiric administration of fomepizole +/- prompt need for dialysis |
| Discuss with nephrology for dialysis  *Scenario time = 11 minutes* | None | Facilitator can prompt “Is there anyone you can call to help treat a suspected toxic alcohol ingestion?” |
| *Admit to Medical ICU*  *Scenario time = 12 minutes* | End Scenario | Learner(s) can be debriefed |

**Ideal Scenario Flow**

- Initial stabilization, including starting IV access, initiating IVF bolus, placing the patient on a cardiac and pulse oximeter monitors
- Check blood sugar on AMS patient as soon as realized
- Intubate patient with depressed mental status (GCS 7) as soon as realized
- Check appropriate placement of ETT immediately after intubation
- Start IV bicarbonate bolus and drip on recognition of profound acidemia
- Troubleshoot etiology of AGMA
- Start fomepizole, consult with Poison Control
- Consult nephrology for dialysis
- Admit the patient to the ICU

The learner(s) start the case with EMS transferring the patient to the ED bed. No history is obtainable from the patient secondary to AMS (GCS 7) and a brief history is available from EMS. The learner(s) should initiate an evaluation of airway, breathing, and circulation, to include direction of the nurse to place the patient on a cardiac monitor and pulse oximetry, establish initial vital signs, and initiate IV access. Confirmation of AMS should prompt checking of a bedside blood sugar. Upon recognition of AMS (GCS 7) and/or unresponsiveness, the learner should intubate the patient using rapid sequence intubation (RSI). After stabilization of the airway and performance of a complete examination, the learner(s) should order labs and testing (including CT head, EKG, comprehensive metabolic panel, arterial or venous blood gas, urinalysis, serum acetaminophen level) to evaluate for AMS. The learner(s) will need to further troubleshoot AGMA, with additional laboratory evaluation as needed to narrow the differential (lactic acid level, aspirin level, urine and/or serum ketones, +/- co-oximetry for CO) if not already sent. All laboratory results will need to be interpreted to conclude that a likely etiology of presentation and cause of the AMGA is toxic alcohol ingestion. Depending on the level of the learner, they can be prompted to recall the antidote and need for initiation, or get further treatment recommendations from the Poison Control Center. The Poison Control Center can provide dosing recommendations, as well as other treatment modalities to include bicarbonate bolus and infusion, dialysis, and confirmatory levels (ethylene glycol and methanol). Prompt initiation of treatment with fomepizole and dialysis prior to definitive diagnosis by confirmatory levels is paramount to the proper management of this type of ingestion. Other learning opportunities in the case include how to mix a bicarbonate drip (150 mEq sodium bicarbonate in 5% Dextrose in Water (D_5_W)) that can be administered for the profound acidemia, as well as to decrease toxicity of formate. Many learners have never ordered toxic alcohol levels, and can be instructed on how to properly order these tests. For more advanced learners, the importance of co-factor therapy (thiamine and pyridoxine for ethylene glycol, folate for methanol), can be stressed. Our learners typically intubated the patient prior to the availability of the initial ABG results. However, the results can prompt a discussion about the challenges when performing RSI given this degree of acidemia, and management options to help mitigate worsening acidemia (use of short acting depolarizing agent, the most experienced clinician preforms the intubation, using bicarbonate bolus (es) just prior to intubation). Additionally, recognition of the need to increase the minute ventilation thru proper ventilator settings (appropriately hyperventilate the patient in an attempt to match the patient’s compensatory drive) can be stressed by review of the pre- and post- intubation ABG. The scenario will end in 12- 15 minutes, depending on the pace of the learner.

**Anticipated Management Mistakes**

1. *Failure to appropriately troubleshoot AGMA:* Although all learners could recount a differential of AGMA when prompted, some learners did not thoroughly troubleshoot the AGMA by addition of other laboratory studies (lactic acid level, consideration of ketosis) as a means to narrowing the differential.
2. *Failure to empirically initiate therapeutic interventions:* This was very common mistake across all learner levels. As toxic alcohol levels are not available during the case, the decision to initiate definite management must be made without test results. Even at institutions that have the capability to test for toxic alcohol levels, the process can still take several hours, and treatment decisions must be made with incomplete information. Learners specifically reported unfamiliarity and/or diagnostic uncertainty with this: “I’ve never seen this type of patient in the ED” or “I am not comfortable administering the antidote without a definitive test result.” Learners would try to admit the patient to the ICU before initiating definitive treatment interventions (fomepizole and dialysis) or defer initiation of management until the patient is admitted to the ICU. This is a great opportunity to empower learners to administer patient care and to re-iterate the importance of not delaying patient care to critically ill patients.
3. *Failure to follow thru with specific recommendations given by the Poison Control Center:* After speaking to the PCC, many learners could not carry out specific recommendation because they could not recount exactly what instructions were given. PCC instructions can be specific and lengthy for toxic alcohol ingestions, including dosing recommendations for fomepizole (dosing and frequency), cofactor therapy (dosing and frequency), initiation of bicarbonate bolus and drip (mixing instructions and rate of administration), need to consult nephrology for dialysis, and need to send toxic alcohol levels. Depending on how much guidance the facilitator gives to the learner, many learners may not be prepared for the volume of instructions. Additionally, graduating residents could often not remember the antidote by name (fomepizole), which may be needed during board examination processes.
4. *Failure to know appropriate way to mix a bicarbonate drip:* As residents heavily rely on ED pharmacists at our institution, many did not know how to mix a bicarbonate drip. This is well within the expected knowledge base for a senior resident, and is an opportunity for education.
5. *Failure to appropriately order toxic alcohol levels*: Many resident have never ordered these before, and have lack of familiarity with the ordering process.
6. *Lack of recognition to adjust ventilator settings:* Many residents overlook the initial ABG (even when prompted by the facilitator) and fail to recognize the need to adjust the ventilator settings to appropriately compensate for the profound acidosis.
7. *Attempt to decontaminate:* Decontamination effort with oral charcoal is not indicated secondary to AMS and unknown time to ingestion.
