# Supplementary material for: Toxic Alcohol Ingestion/Methanol Ingestion
Source: MedEdPORTAL. 2018 Aug 17;14:10740. doi: 10.15766/mep_2374-8265.10740 (PMC6342378; doi:10.15766/mep_2374-8265.10740)
Supplement: Supplementary file 1 — A. Methanol Simulation Case.docx B. Methanol Supplemental Case Findings.ppt C. Methanol Questionnaire.docx D. Methanol Evaluation Form.doc E. Methanol Case Debriefing.pptx [file mep-14-10740-s001.zip › C._Methanol_Questionnaire.docx]

Toxic Alcohol/Methanol Educational Event Questionnaire

| Rate how strongly you agree or disagree with each of the following statements following the educational event. Provide written comments if desired. | | | | | |
| --- | --- | --- | --- | --- | --- |
|  | Strongly Disagree | Disagree | Neutral | Agree | Strongly Agree |
| Overall, I am satisfied with this educational event |  |  |  |  |  |
| I am better equipped to identify the signs and symptoms of a toxic alcohol/methanol intoxication |  |  |  |  |  |
| I am more confident in my ability to critically evaluate causes of acid-base disturbances |  |  |  |  |  |
| I have better understanding of the treatment goals for this poisoning and specific indications for management options |  |  |  |  |  |
| This event enhanced my knowledge of methanol toxicity |  |  |  |  |  |
| The debrief was effective in presenting the educational objectives |  |  |  |  |  |
| Comments: | | | | | |
