# Supplementary material for: Toxic Alcohol Ingestion/Methanol Ingestion
Source: MedEdPORTAL. 2018 Aug 17;14:10740. doi: 10.15766/mep_2374-8265.10740 (PMC6342378; doi:10.15766/mep_2374-8265.10740)
Supplement: Supplementary file 1 — A. Methanol Simulation Case.docx B. Methanol Supplemental Case Findings.ppt C. Methanol Questionnaire.docx D. Methanol Evaluation Form.doc E. Methanol Case Debriefing.pptx [file mep-14-10740-s001.zip › E._Methanol_Case_Debriefing.pptx]

## Slide 1
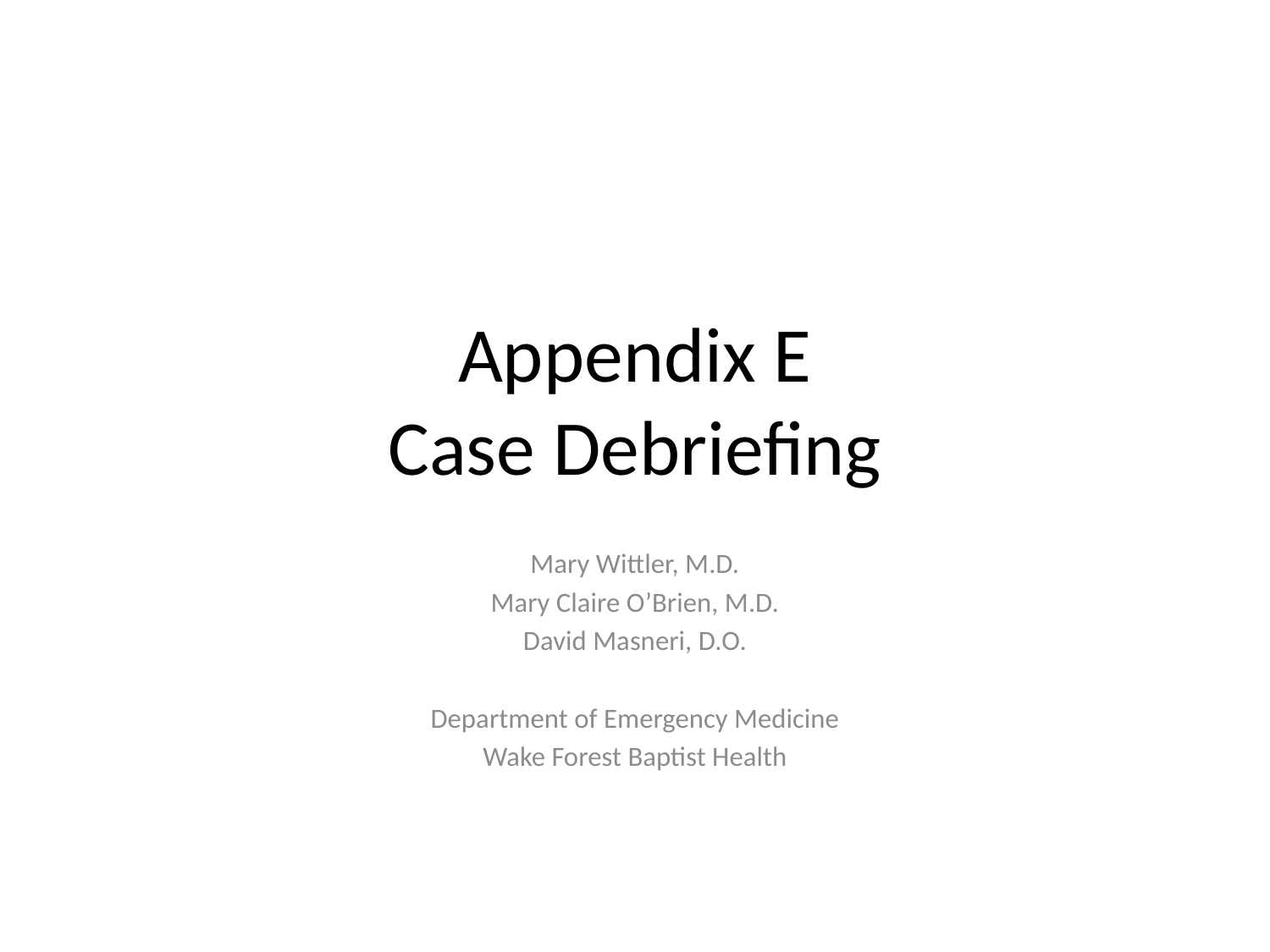

# Appendix E Case Debriefing
Mary Wittler, M.D.
Mary Claire O’Brien, M.D.
David Masneri, D.O.
Department of Emergency Medicine
Wake Forest Baptist Health

## Slide 2
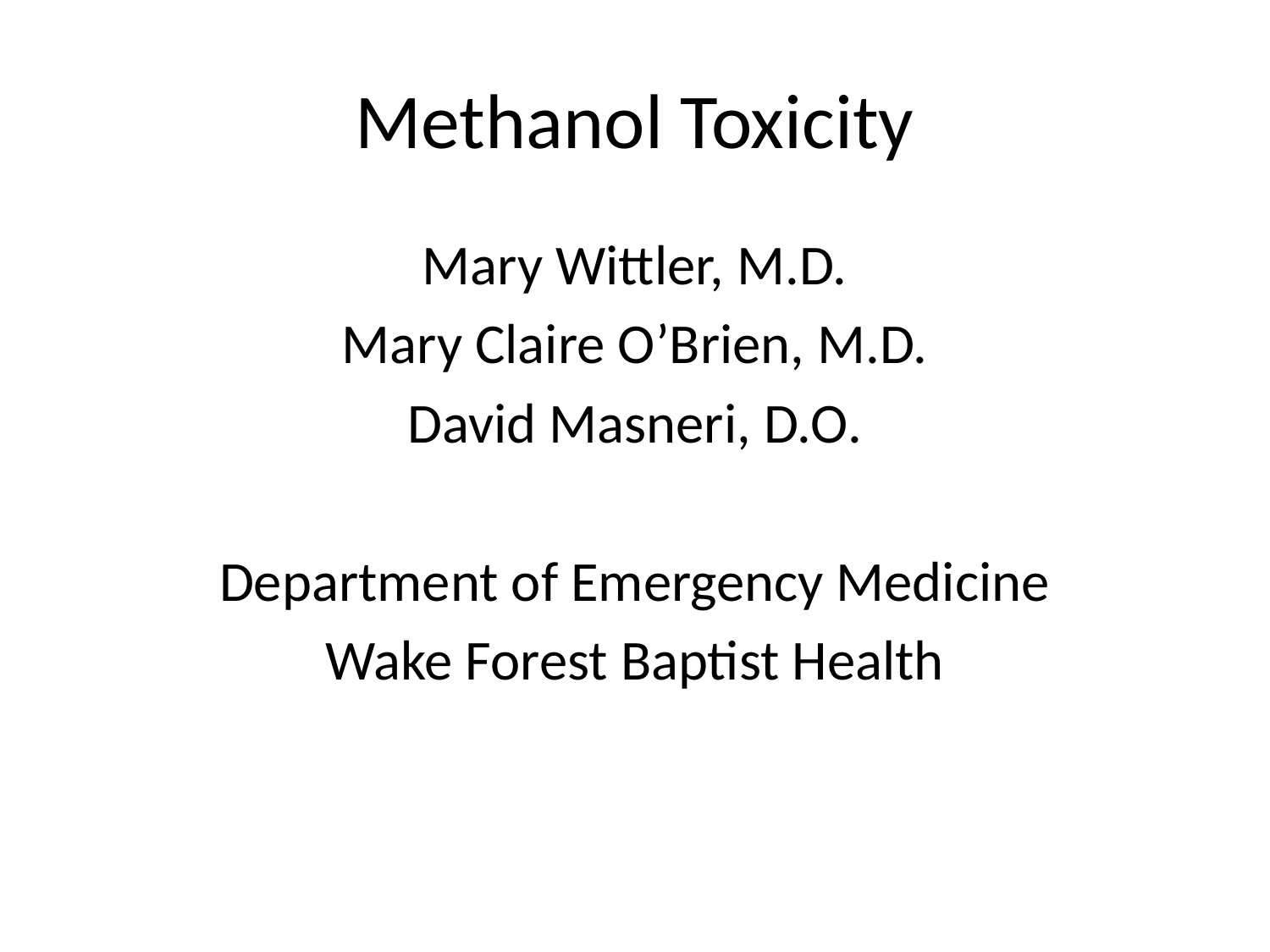

# Methanol Toxicity
Mary Wittler, M.D.
Mary Claire O’Brien, M.D.
David Masneri, D.O.
Department of Emergency Medicine
Wake Forest Baptist Health

## Slide 3
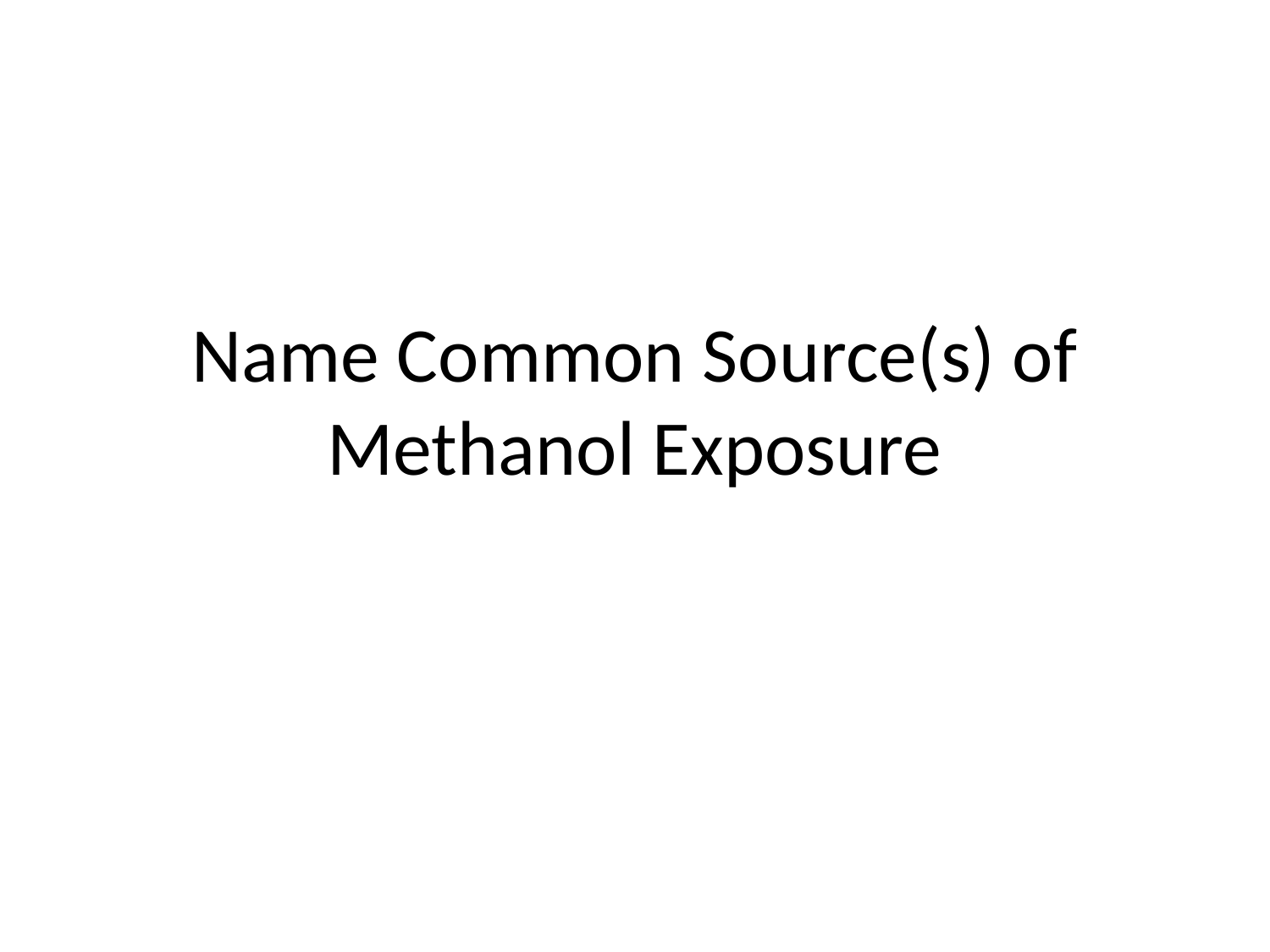

# Name Common Source(s) of Methanol Exposure

## Slide 4
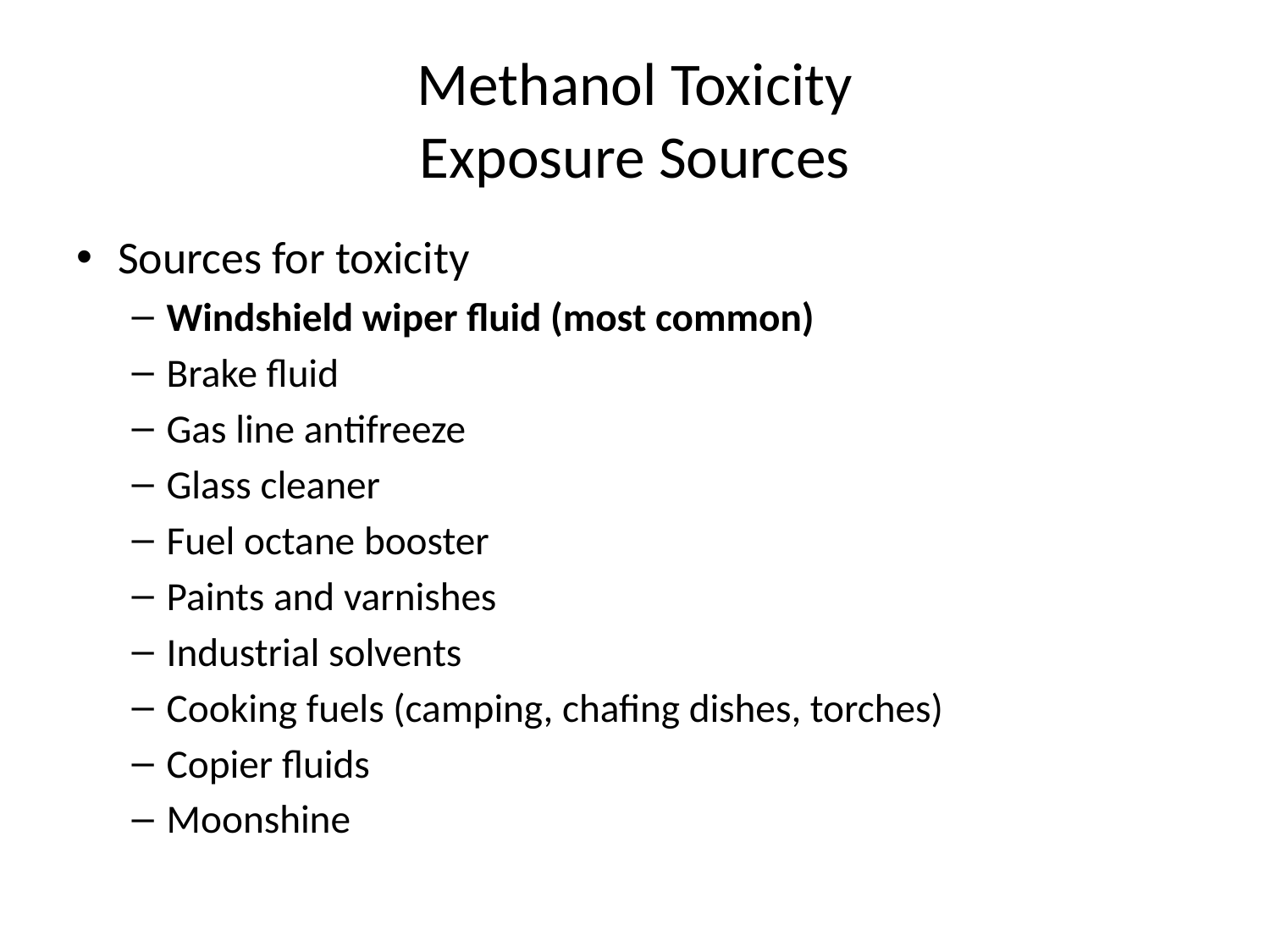

# Methanol Toxicity Exposure Sources
Sources for toxicity
Windshield wiper fluid (most common)
Brake fluid
Gas line antifreeze
Glass cleaner
Fuel octane booster
Paints and varnishes
Industrial solvents
Cooking fuels (camping, chafing dishes, torches)
Copier fluids
Moonshine

## Slide 5
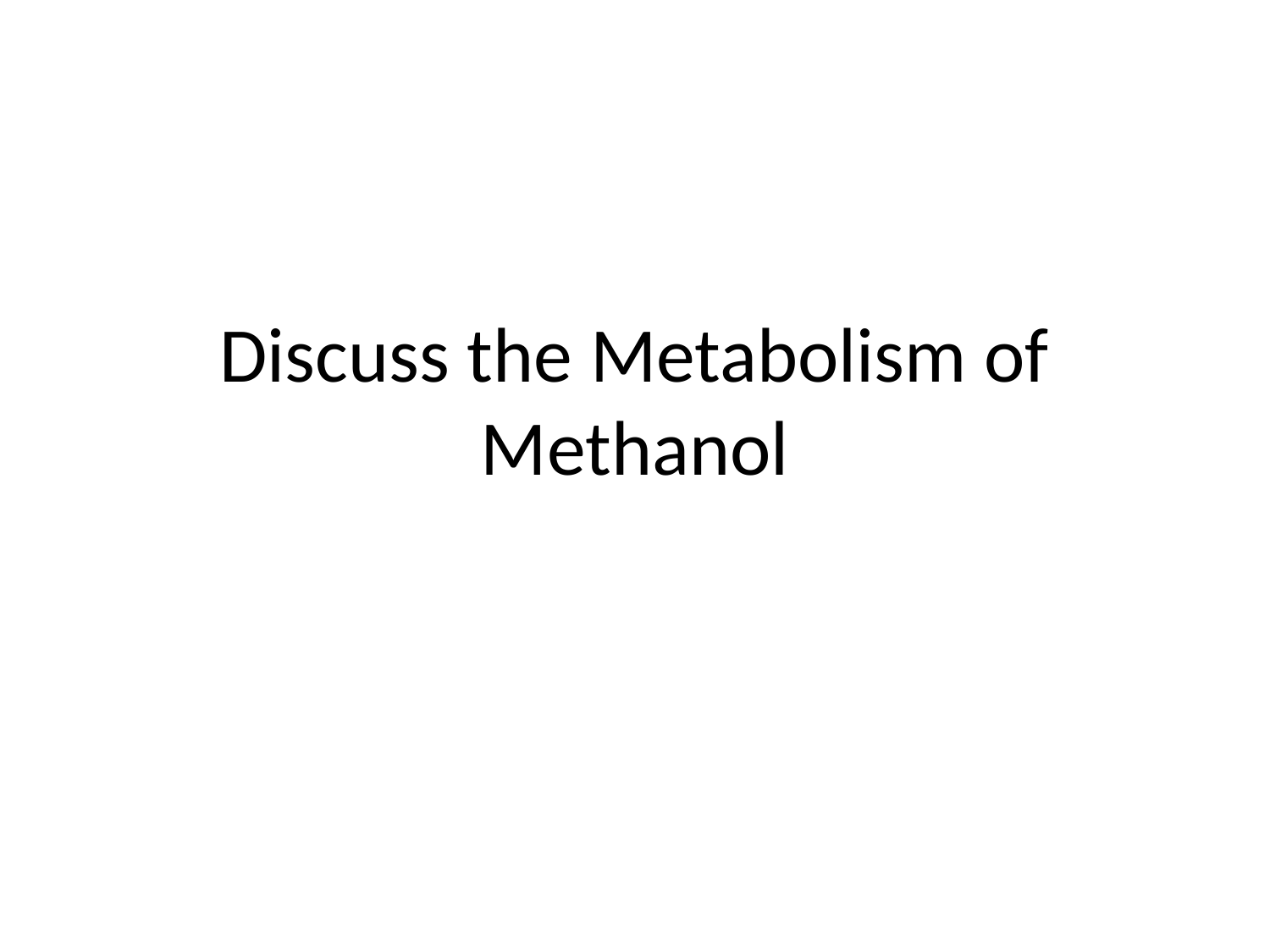

# Discuss the Metabolism of Methanol

## Slide 6
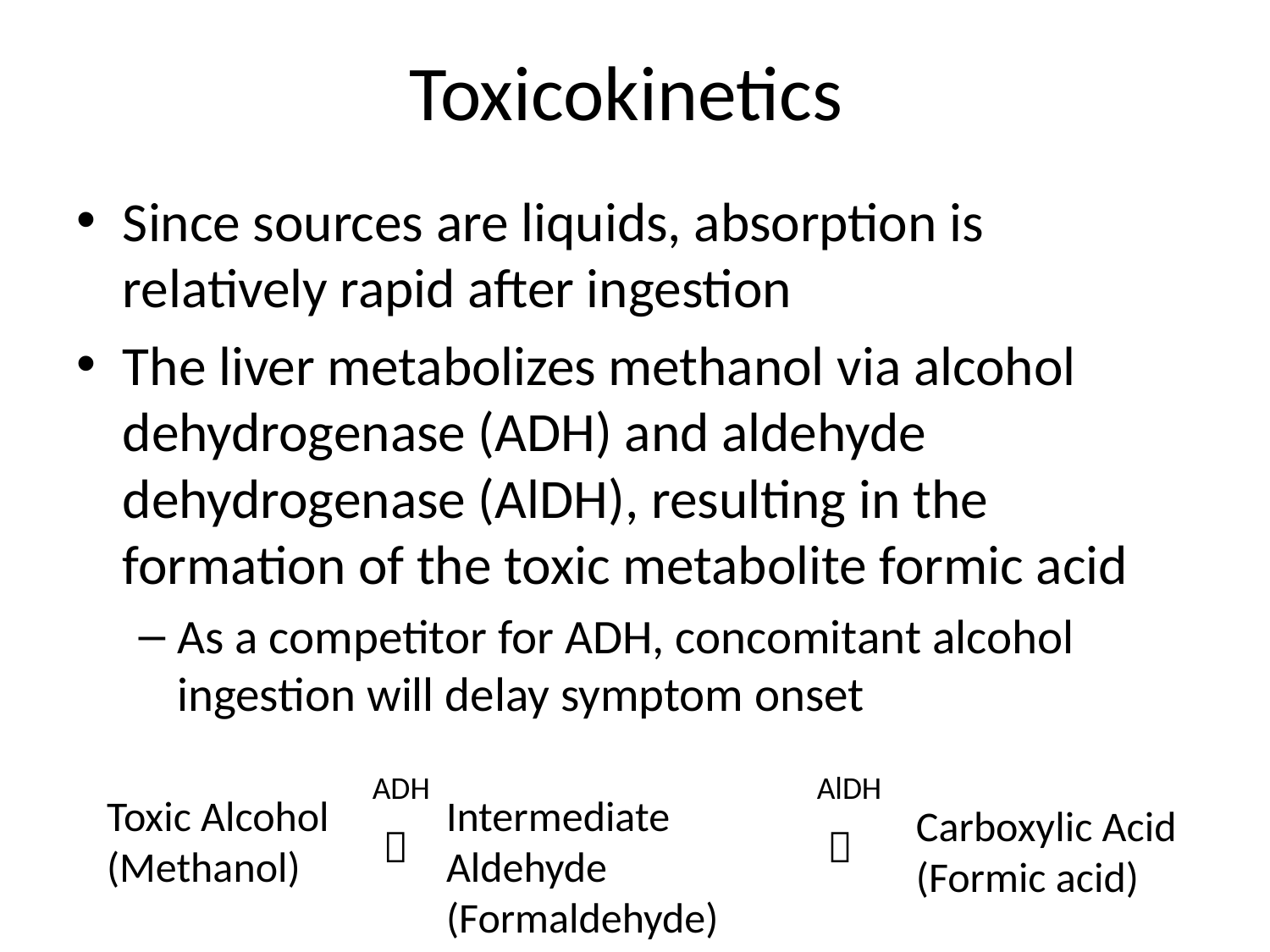

# Toxicokinetics
Since sources are liquids, absorption is relatively rapid after ingestion
The liver metabolizes methanol via alcohol dehydrogenase (ADH) and aldehyde dehydrogenase (AlDH), resulting in the formation of the toxic metabolite formic acid
As a competitor for ADH, concomitant alcohol ingestion will delay symptom onset
ADH
AlDH
Toxic Alcohol
(Methanol)
Intermediate Aldehyde
(Formaldehyde)
Carboxylic Acid
(Formic acid)



## Slide 7
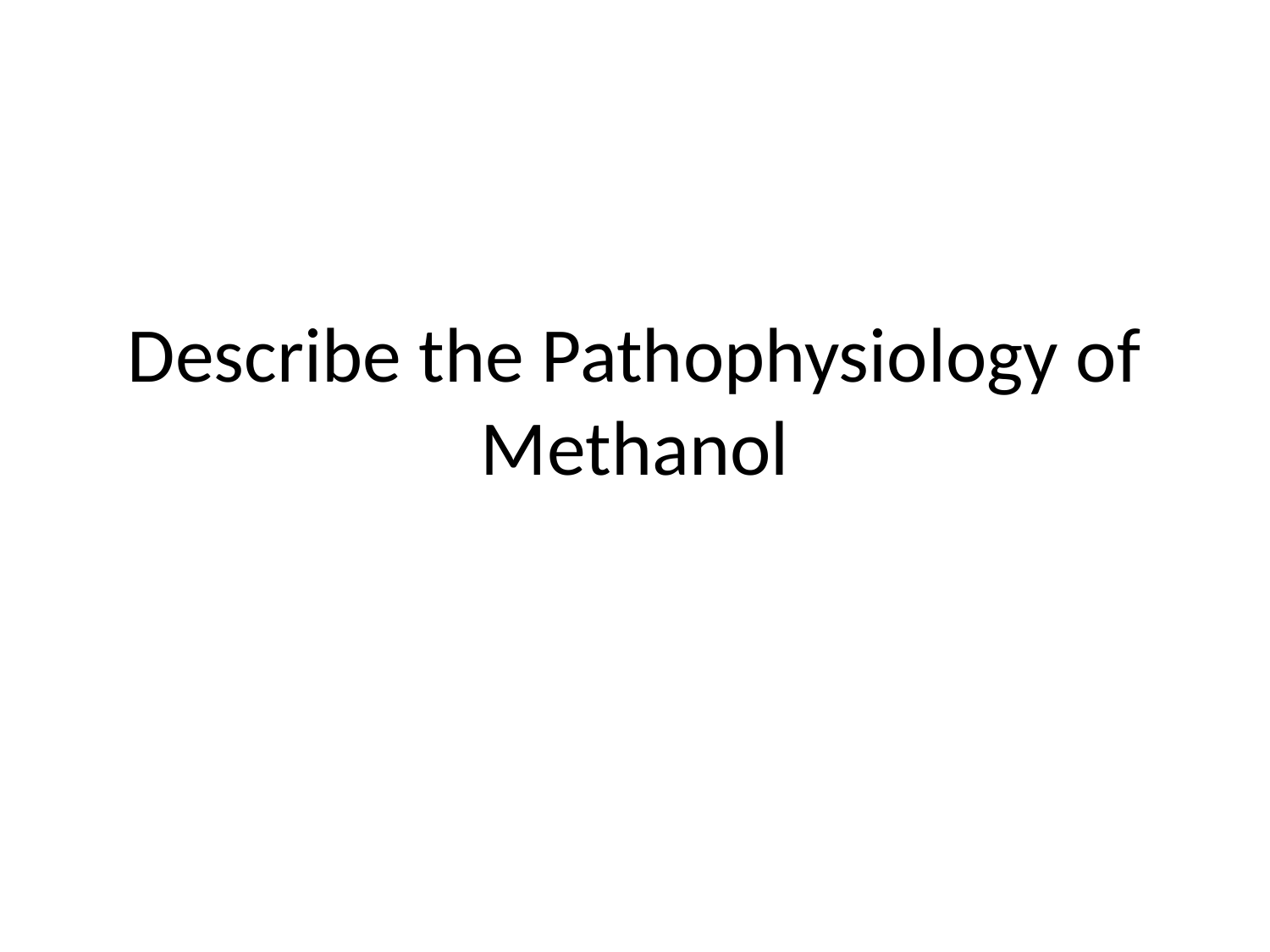

# Describe the Pathophysiology of Methanol

## Slide 8
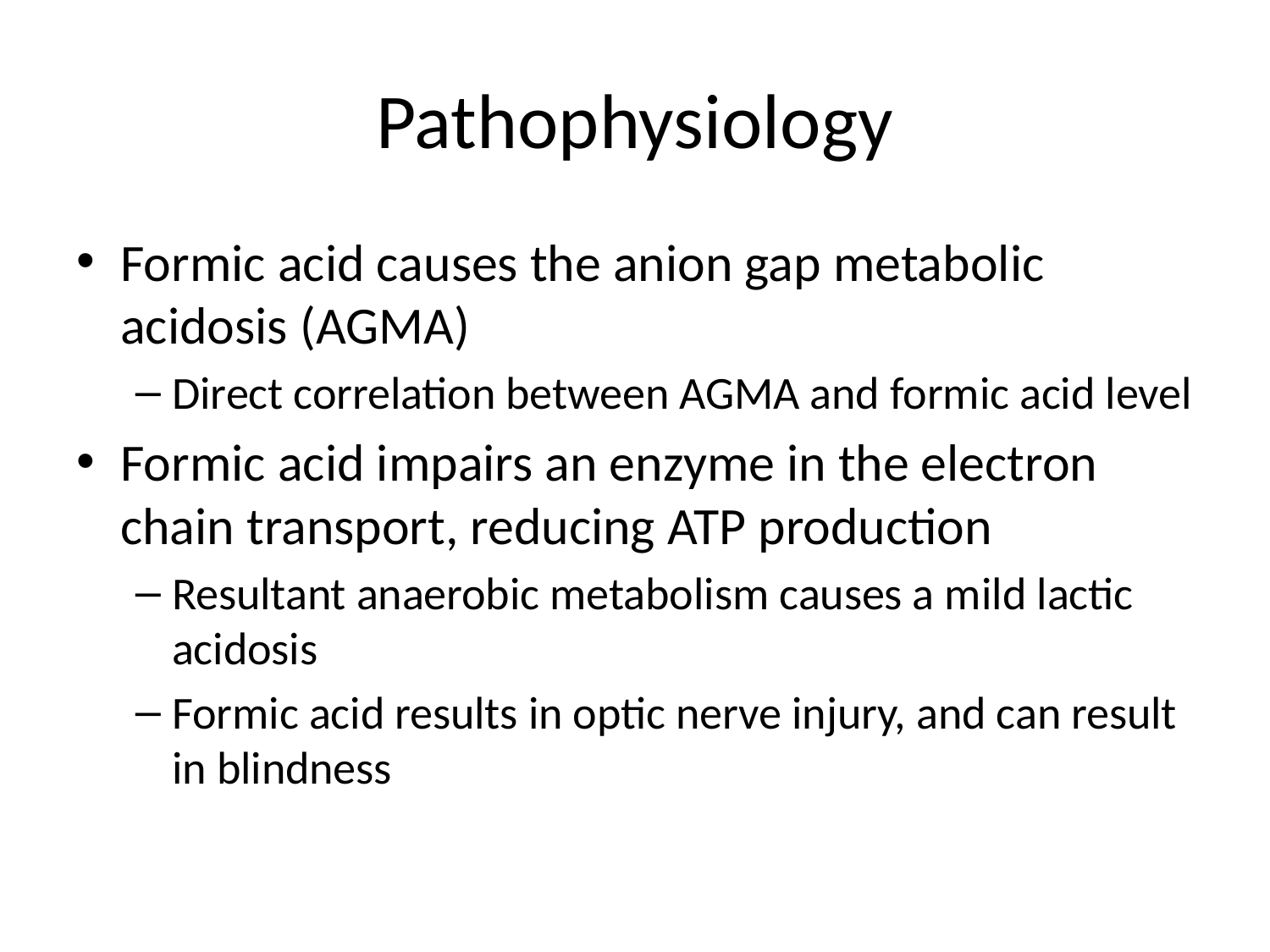

# Pathophysiology
Formic acid causes the anion gap metabolic acidosis (AGMA)
Direct correlation between AGMA and formic acid level
Formic acid impairs an enzyme in the electron chain transport, reducing ATP production
Resultant anaerobic metabolism causes a mild lactic acidosis
Formic acid results in optic nerve injury, and can result in blindness

## Slide 9
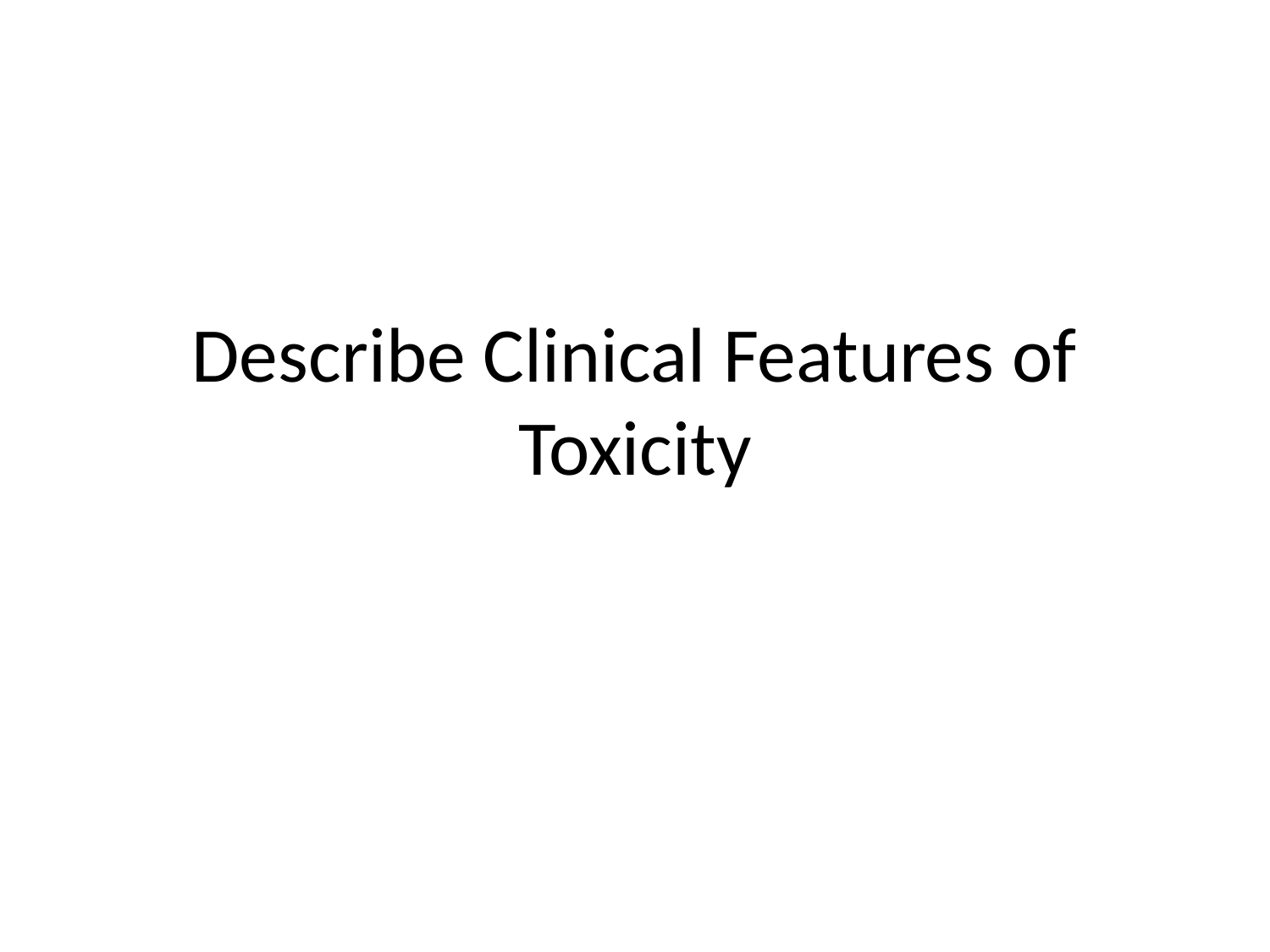

# Describe Clinical Features of Toxicity

## Slide 10
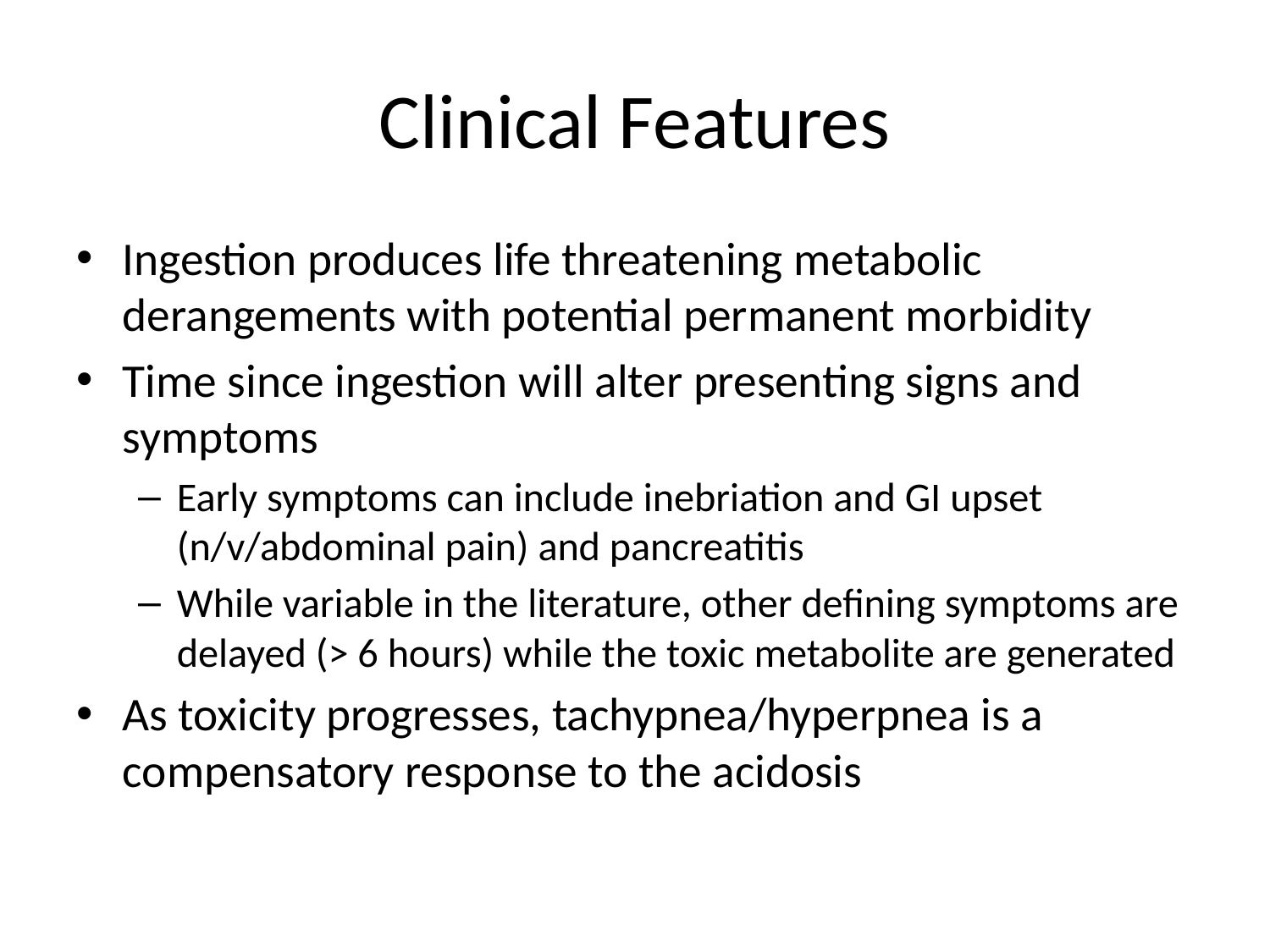

# Clinical Features
Ingestion produces life threatening metabolic derangements with potential permanent morbidity
Time since ingestion will alter presenting signs and symptoms
Early symptoms can include inebriation and GI upset (n/v/abdominal pain) and pancreatitis
While variable in the literature, other defining symptoms are delayed (> 6 hours) while the toxic metabolite are generated
As toxicity progresses, tachypnea/hyperpnea is a compensatory response to the acidosis

## Slide 11
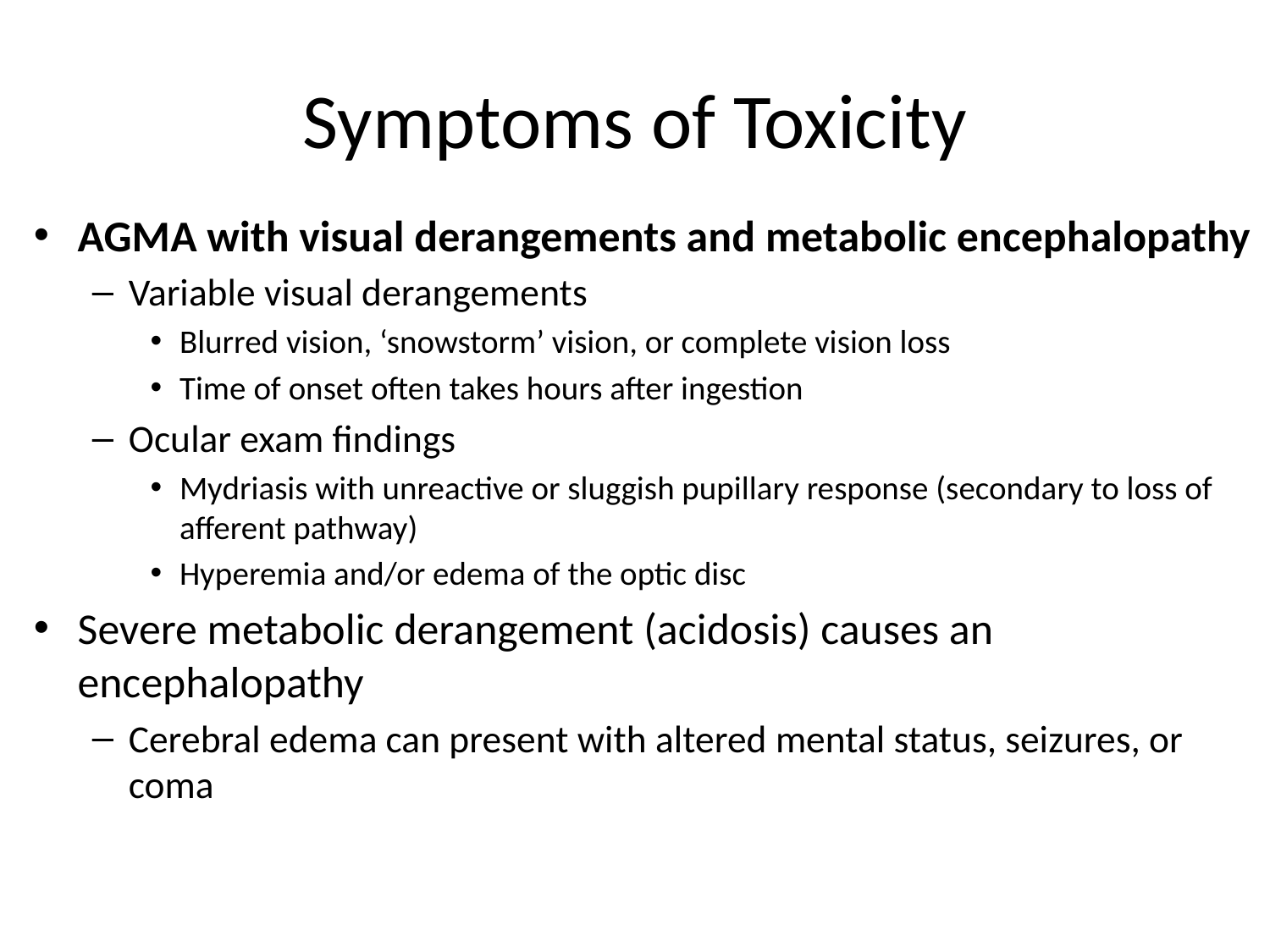

# Symptoms of Toxicity
AGMA with visual derangements and metabolic encephalopathy
Variable visual derangements
Blurred vision, ‘snowstorm’ vision, or complete vision loss
Time of onset often takes hours after ingestion
Ocular exam findings
Mydriasis with unreactive or sluggish pupillary response (secondary to loss of afferent pathway)
Hyperemia and/or edema of the optic disc
Severe metabolic derangement (acidosis) causes an encephalopathy
Cerebral edema can present with altered mental status, seizures, or coma

## Slide 12
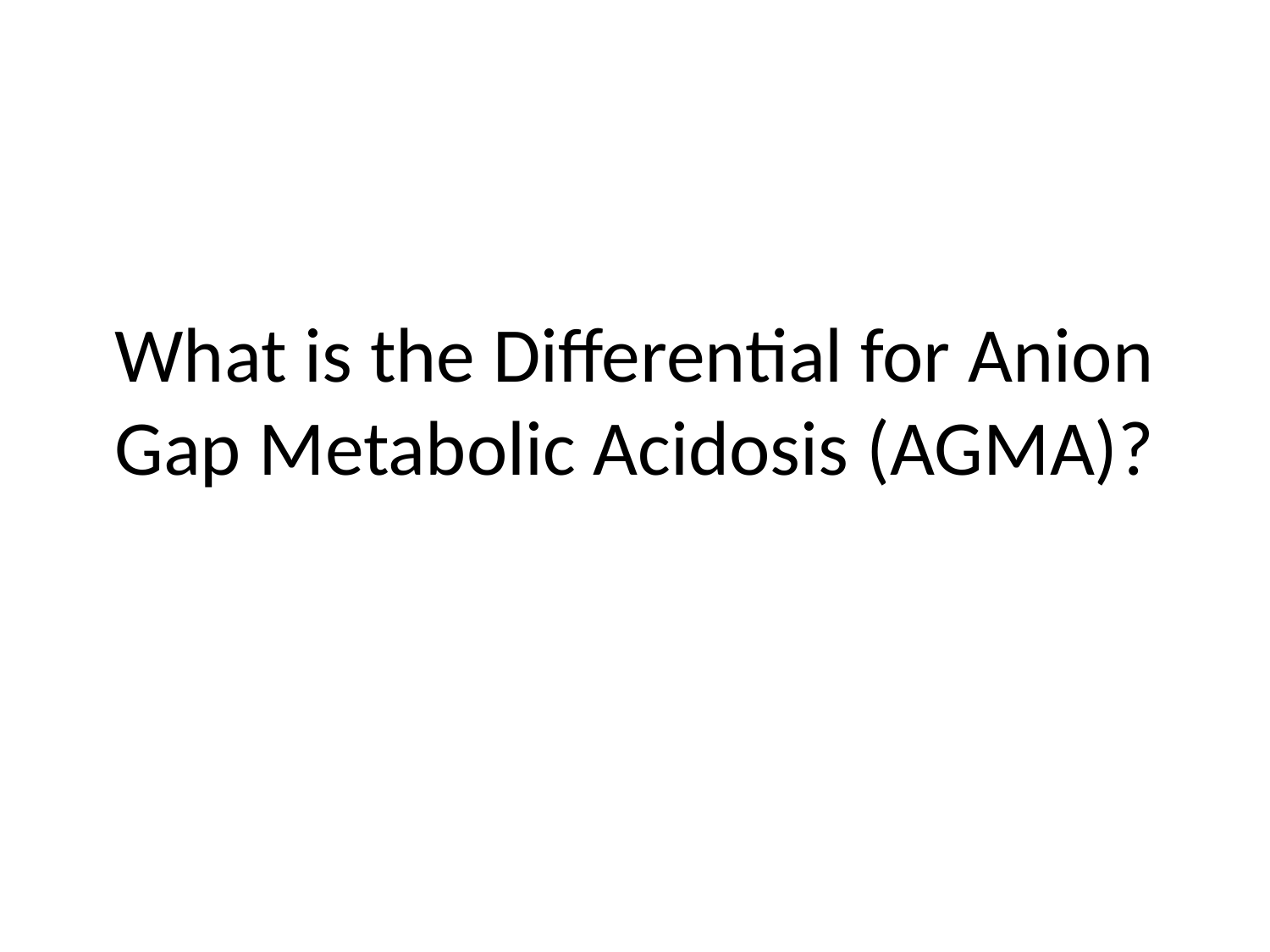

# What is the Differential for Anion Gap Metabolic Acidosis (AGMA)?

## Slide 13
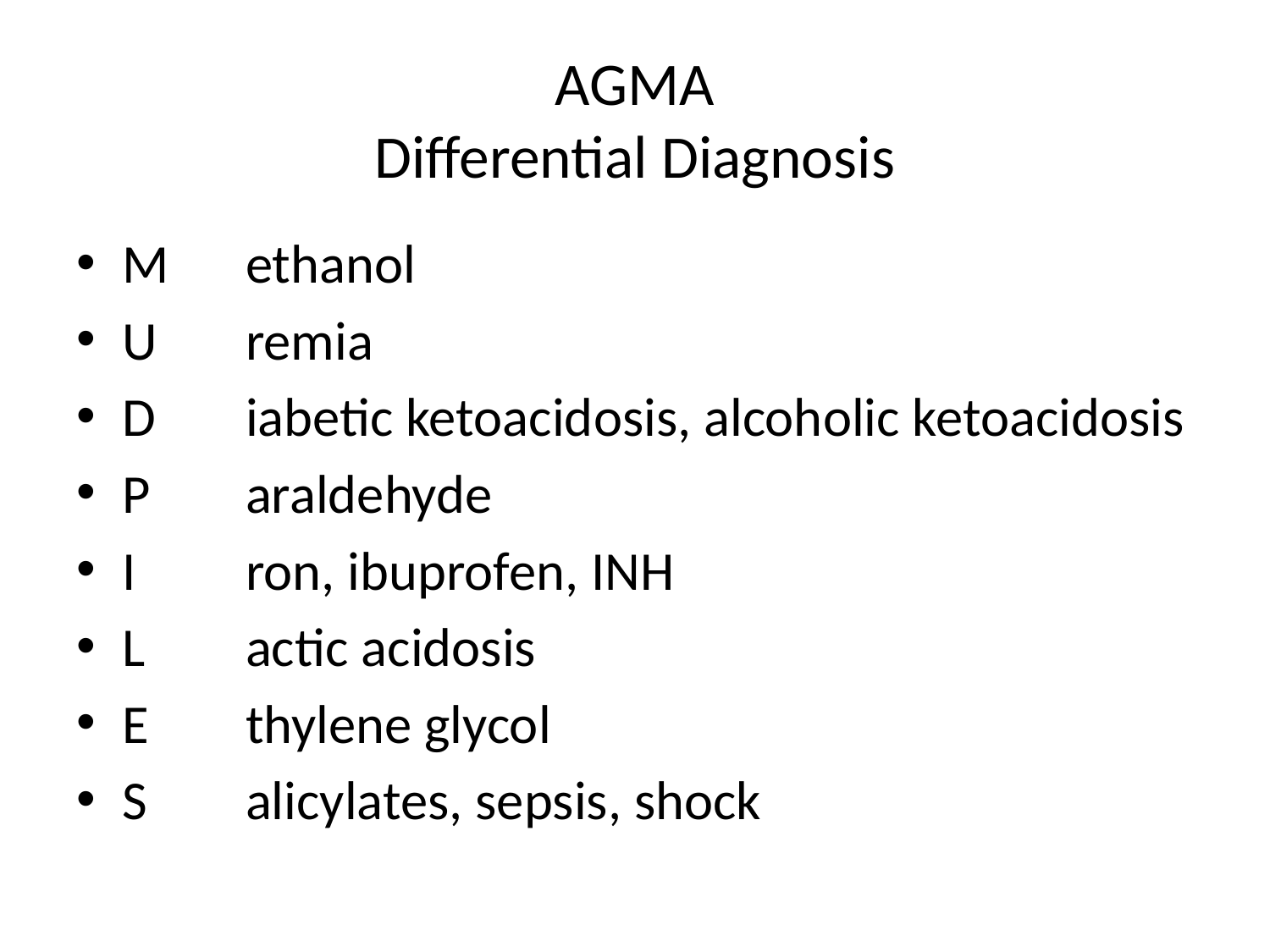

# AGMA Differential Diagnosis
M	ethanol
U	remia
D	iabetic ketoacidosis, alcoholic ketoacidosis
P	araldehyde
I	ron, ibuprofen, INH
L	actic acidosis
E	thylene glycol
S	alicylates, sepsis, shock

## Slide 14
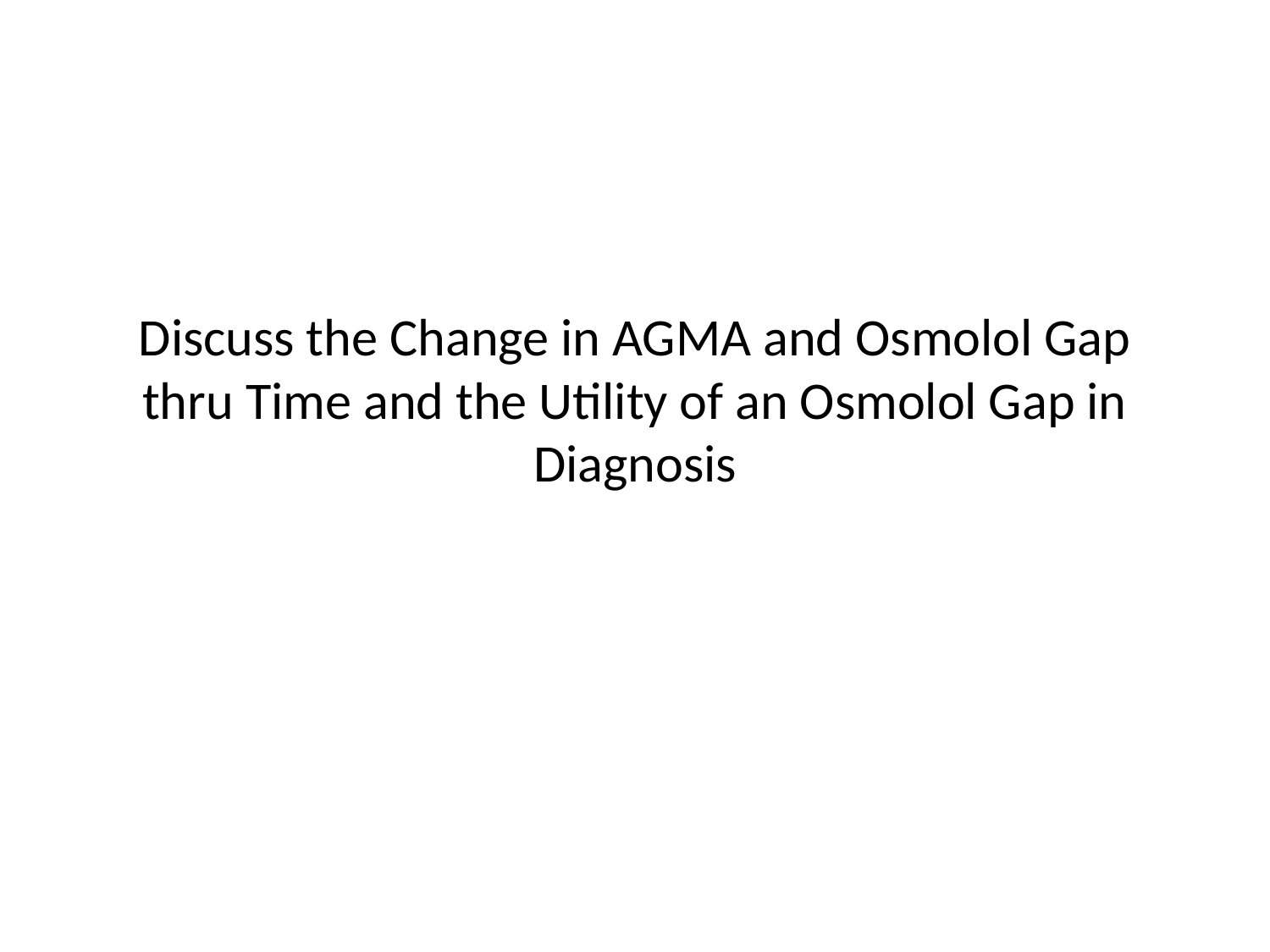

# Discuss the Change in AGMA and Osmolol Gap thru Time and the Utility of an Osmolol Gap in Diagnosis

## Slide 15
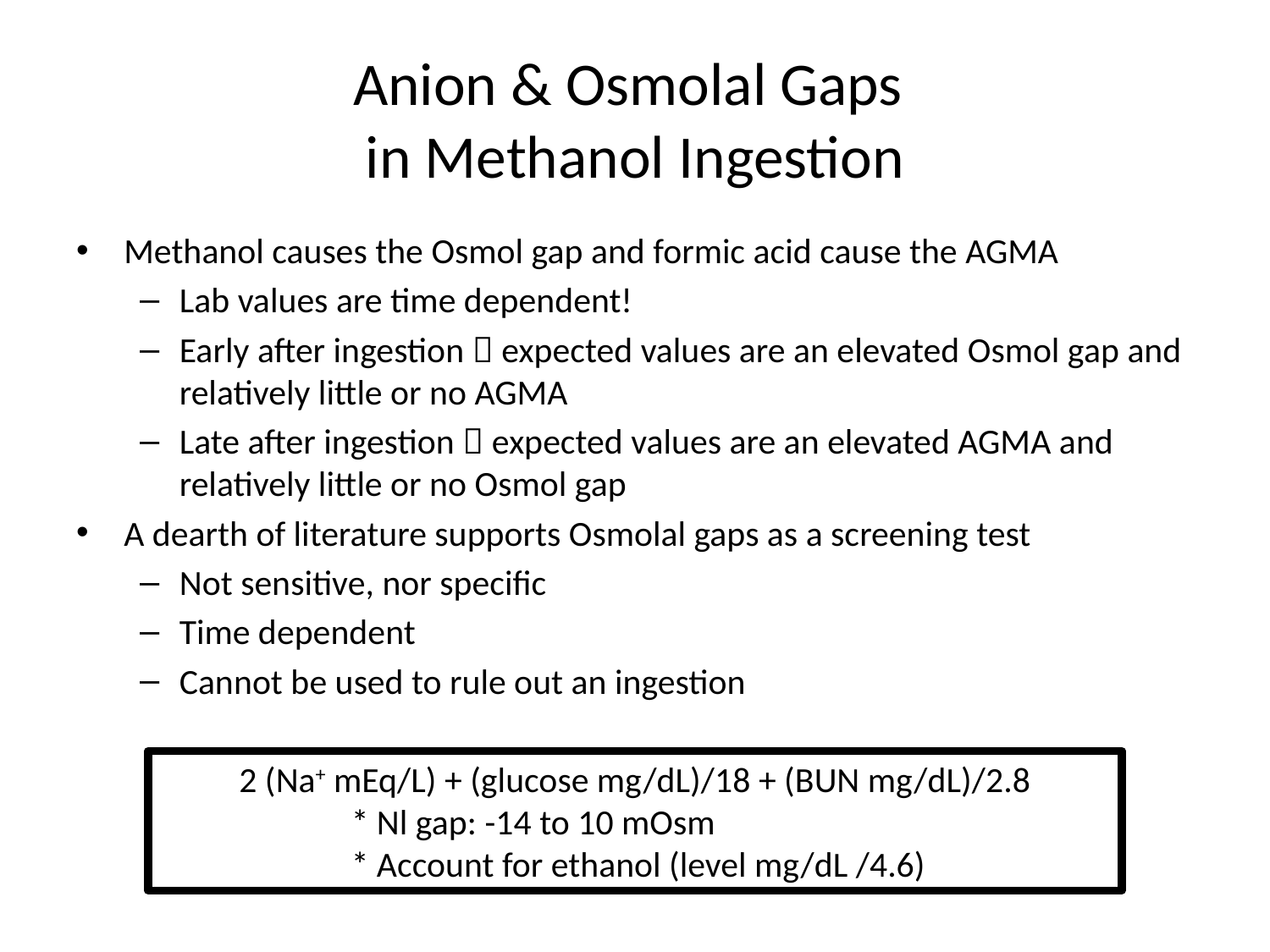

# Anion & Osmolal Gaps in Methanol Ingestion
Methanol causes the Osmol gap and formic acid cause the AGMA
Lab values are time dependent!
Early after ingestion  expected values are an elevated Osmol gap and relatively little or no AGMA
Late after ingestion  expected values are an elevated AGMA and relatively little or no Osmol gap
A dearth of literature supports Osmolal gaps as a screening test
Not sensitive, nor specific
Time dependent
Cannot be used to rule out an ingestion
2 (Na+ mEq/L) + (glucose mg/dL)/18 + (BUN mg/dL)/2.8
	* Nl gap: -14 to 10 mOsm
	* Account for ethanol (level mg/dL /4.6)

## Slide 16
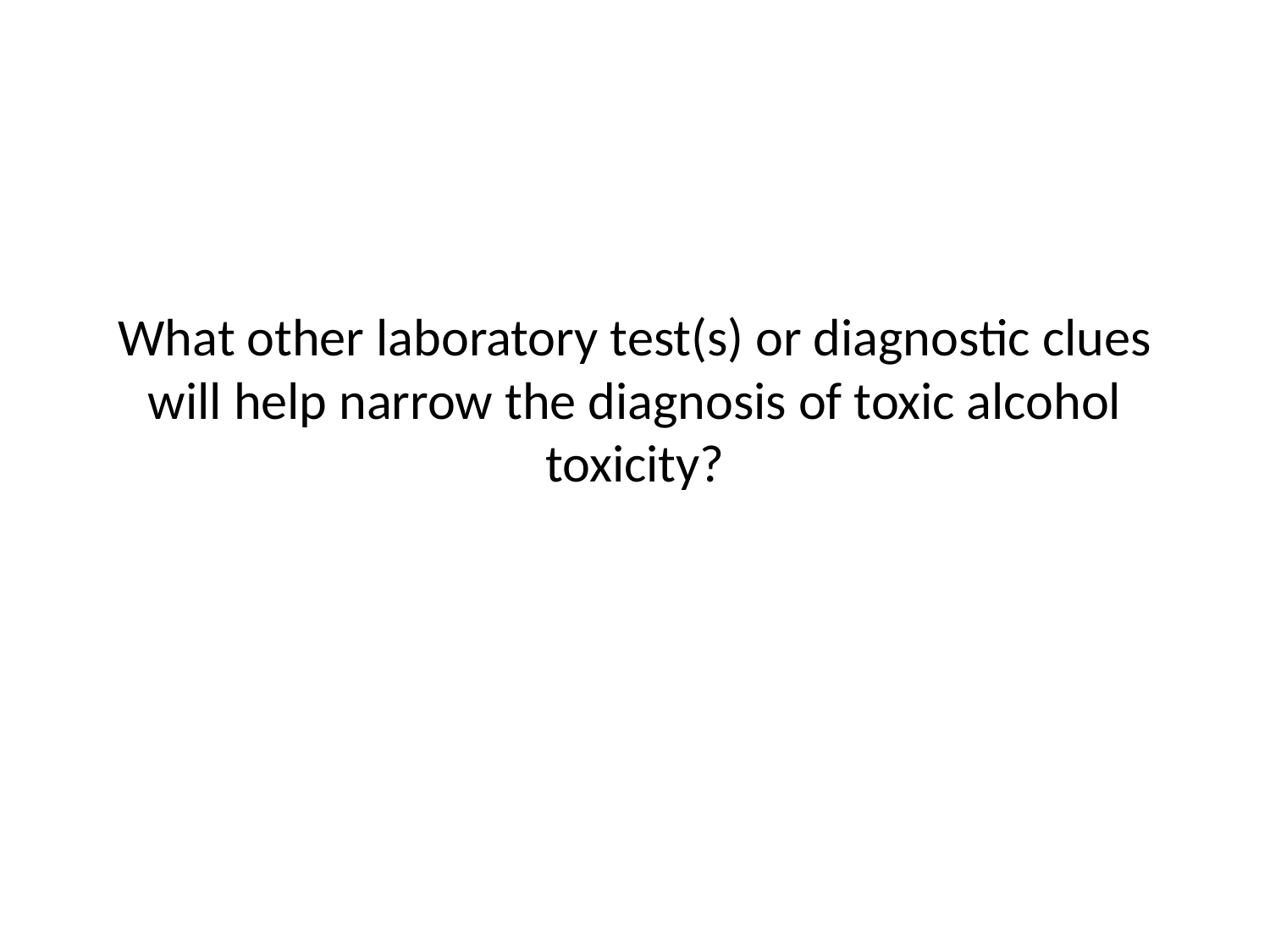

# What other laboratory test(s) or diagnostic clues will help narrow the diagnosis of toxic alcohol toxicity?

## Slide 17
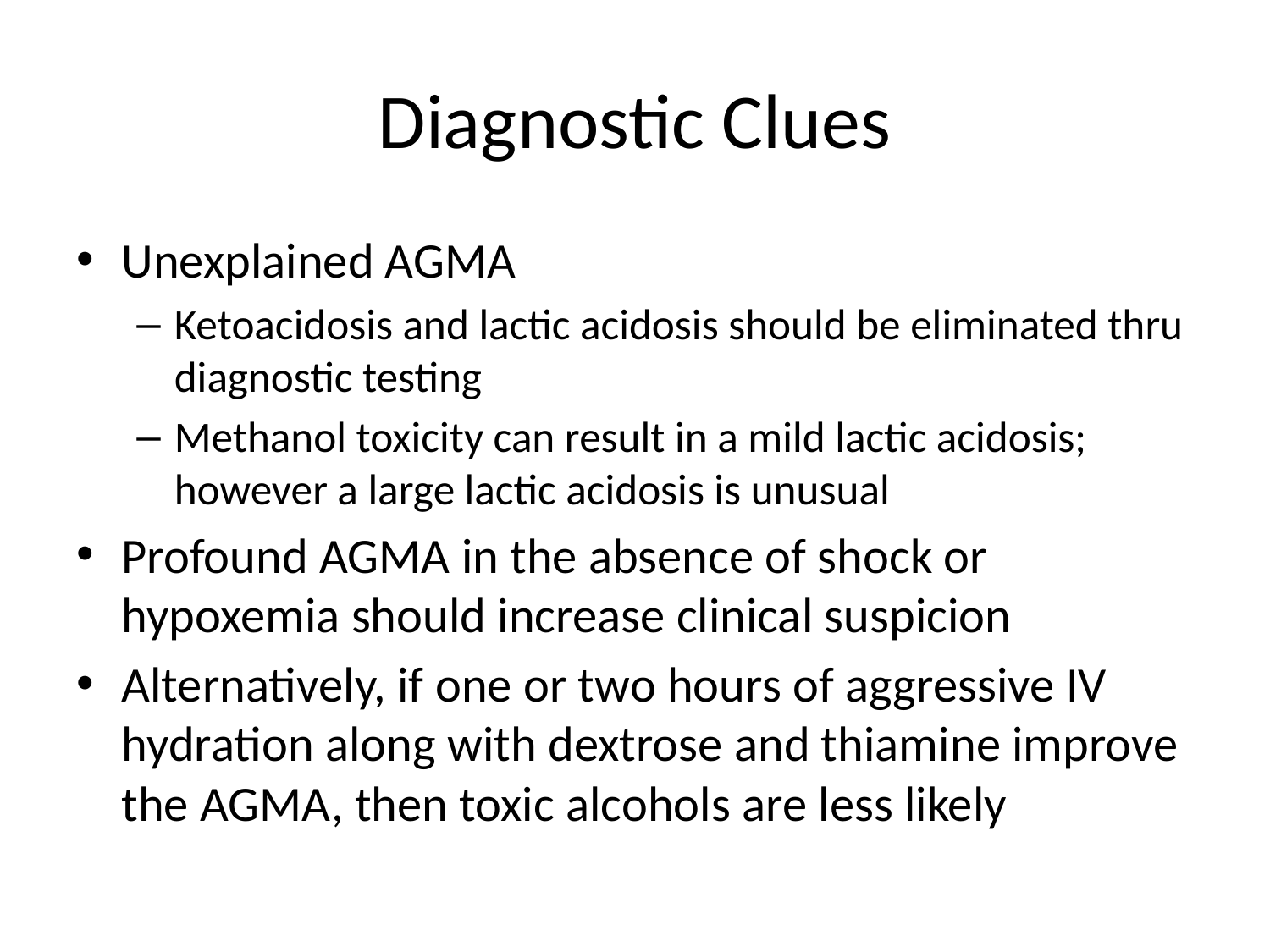

# Diagnostic Clues
Unexplained AGMA
Ketoacidosis and lactic acidosis should be eliminated thru diagnostic testing
Methanol toxicity can result in a mild lactic acidosis; however a large lactic acidosis is unusual
Profound AGMA in the absence of shock or hypoxemia should increase clinical suspicion
Alternatively, if one or two hours of aggressive IV hydration along with dextrose and thiamine improve the AGMA, then toxic alcohols are less likely

## Slide 18
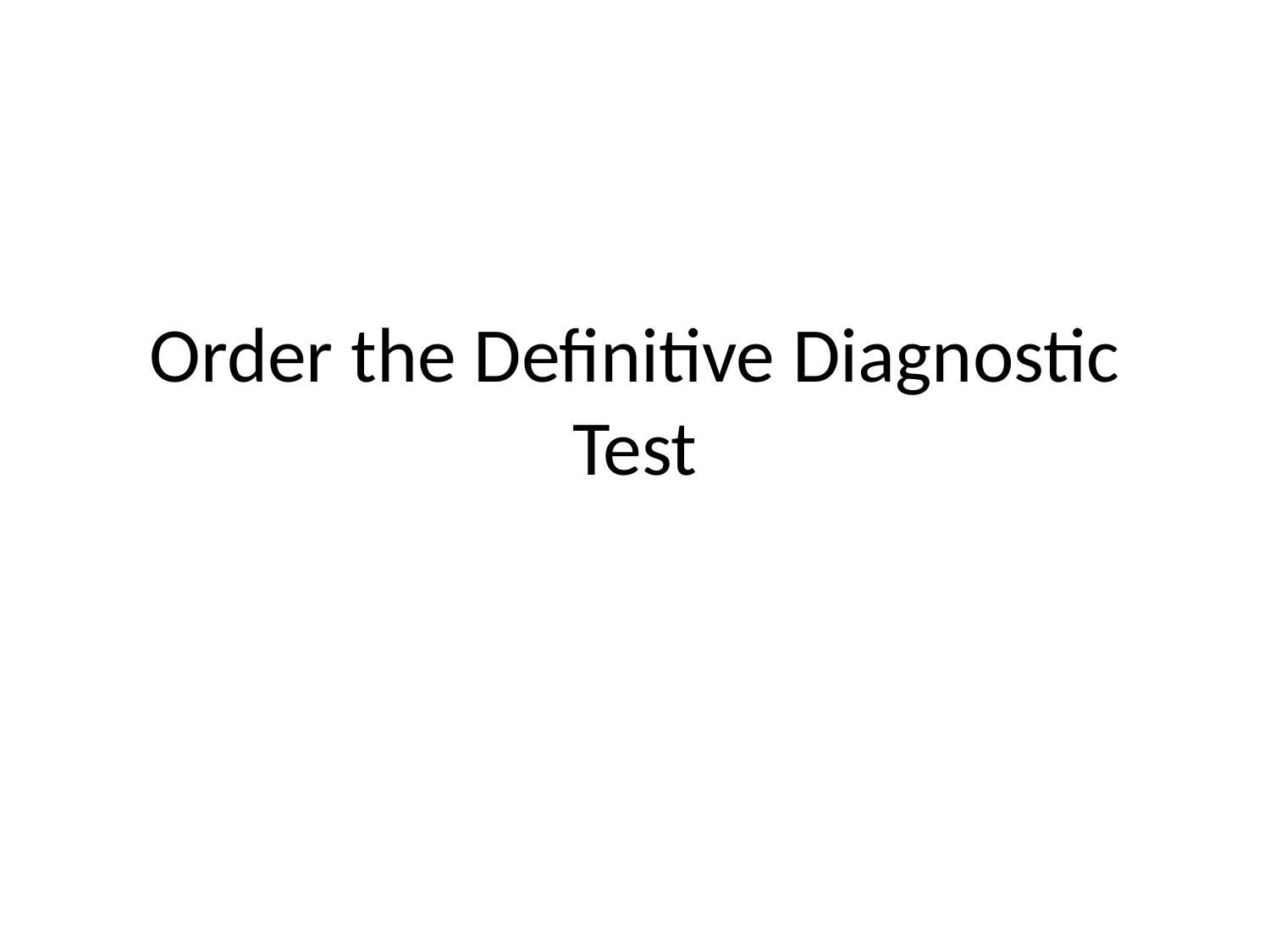

# Order the Definitive Diagnostic Test

## Slide 19
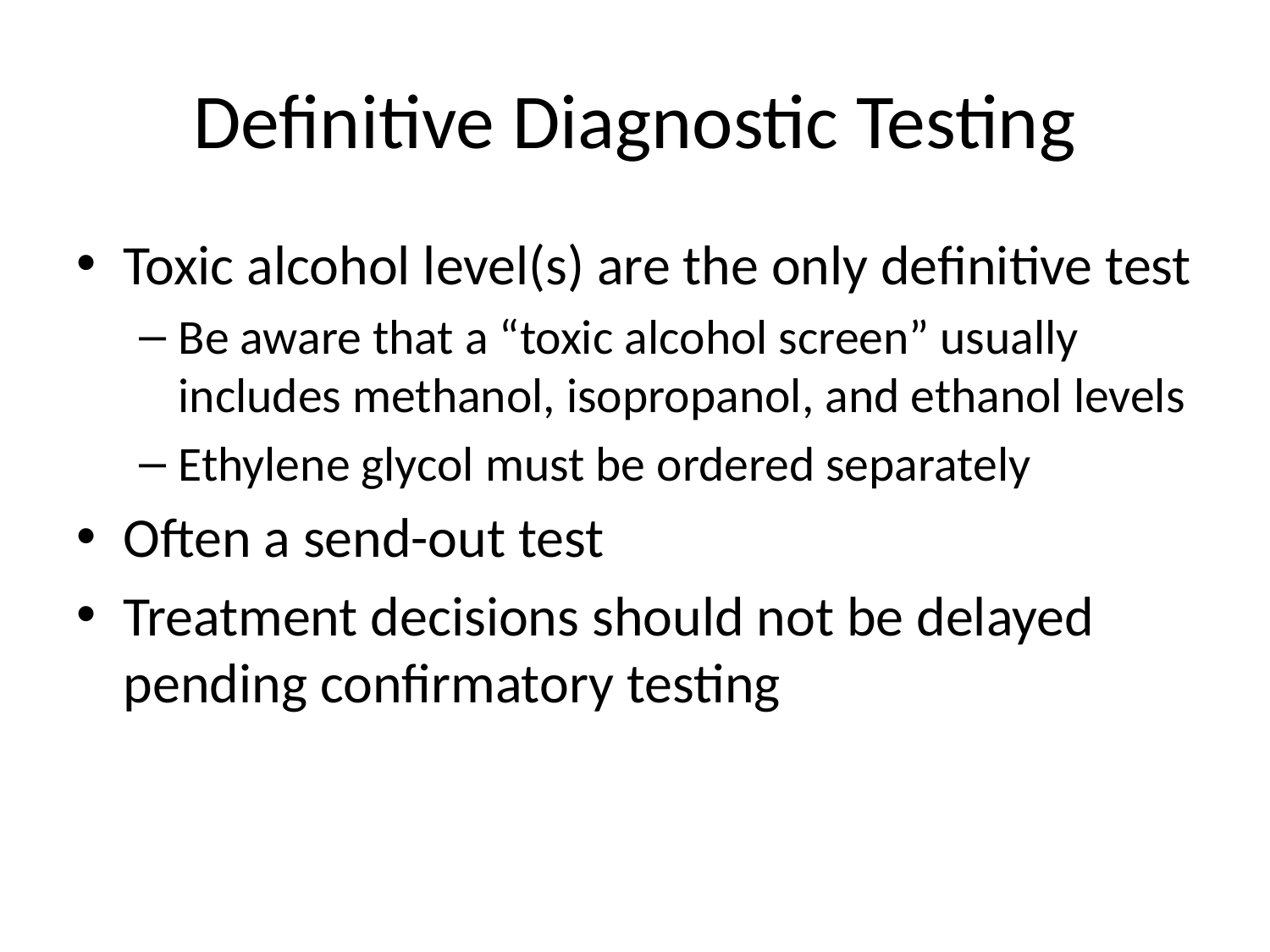

# Definitive Diagnostic Testing
Toxic alcohol level(s) are the only definitive test
Be aware that a “toxic alcohol screen” usually includes methanol, isopropanol, and ethanol levels
Ethylene glycol must be ordered separately
Often a send-out test
Treatment decisions should not be delayed pending confirmatory testing

## Slide 20
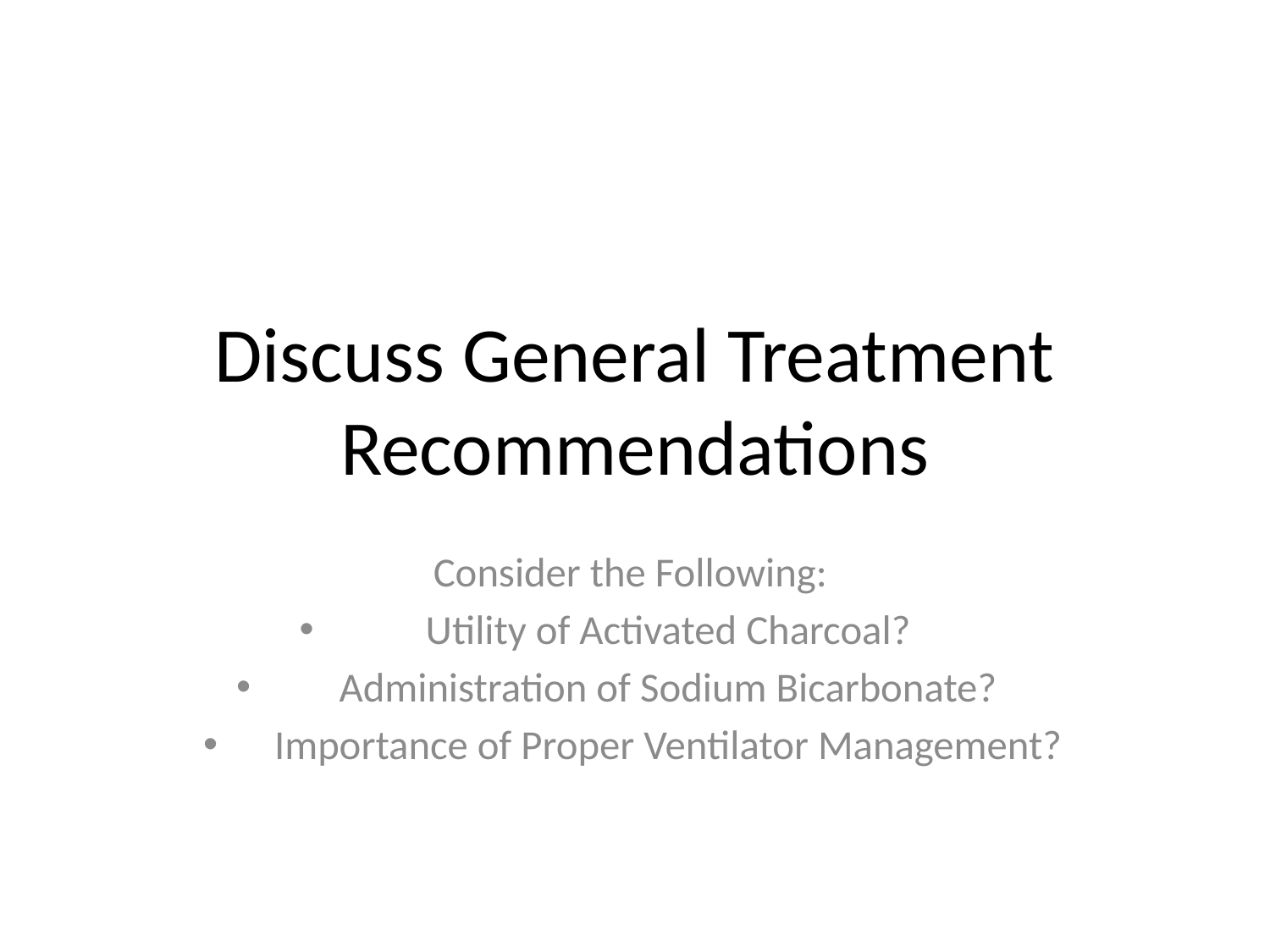

# Discuss General Treatment Recommendations
Consider the Following:
Utility of Activated Charcoal?
Administration of Sodium Bicarbonate?
Importance of Proper Ventilator Management?

## Slide 21
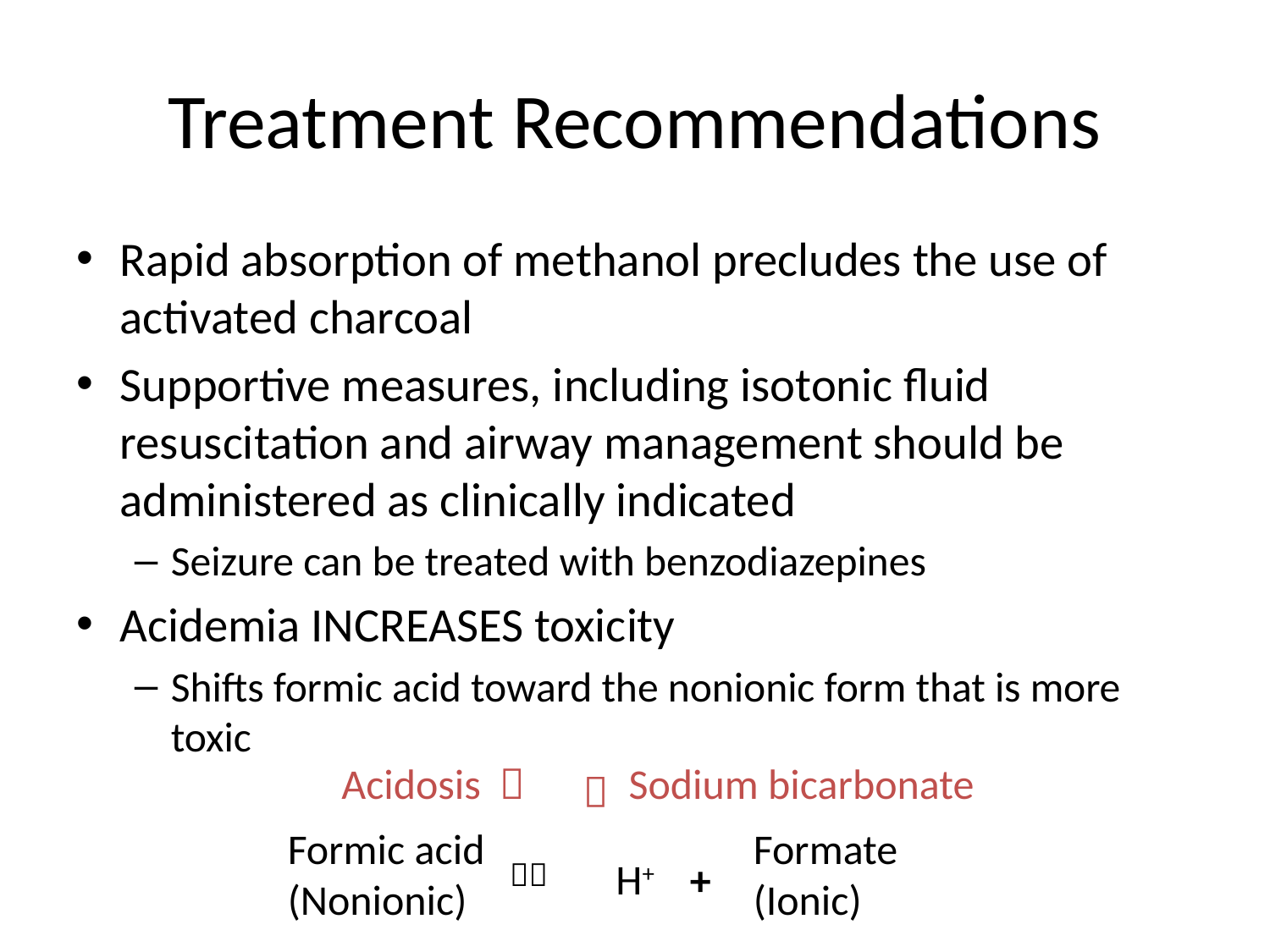

# Treatment Recommendations
Rapid absorption of methanol precludes the use of activated charcoal
Supportive measures, including isotonic fluid resuscitation and airway management should be administered as clinically indicated
Seizure can be treated with benzodiazepines
Acidemia INCREASES toxicity
Shifts formic acid toward the nonionic form that is more toxic
Acidosis

Sodium bicarbonate

Formic acid
(Nonionic)
Formate
(Ionic)
H+
+


## Slide 22
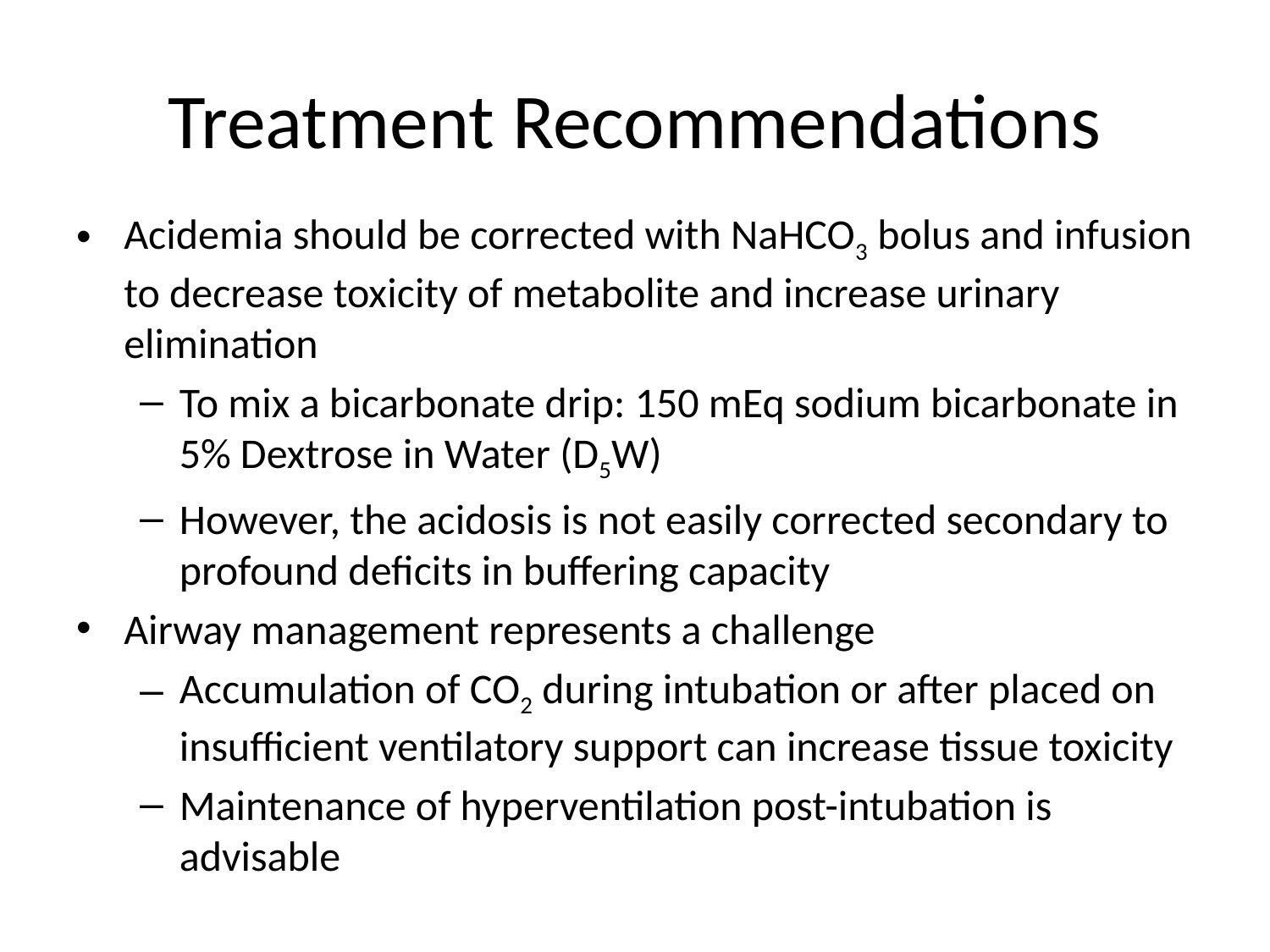

# Treatment Recommendations
Acidemia should be corrected with NaHCO3 bolus and infusion to decrease toxicity of metabolite and increase urinary elimination
To mix a bicarbonate drip: 150 mEq sodium bicarbonate in 5% Dextrose in Water (D5W)
However, the acidosis is not easily corrected secondary to profound deficits in buffering capacity
Airway management represents a challenge
Accumulation of CO2 during intubation or after placed on insufficient ventilatory support can increase tissue toxicity
Maintenance of hyperventilation post-intubation is advisable

## Slide 23
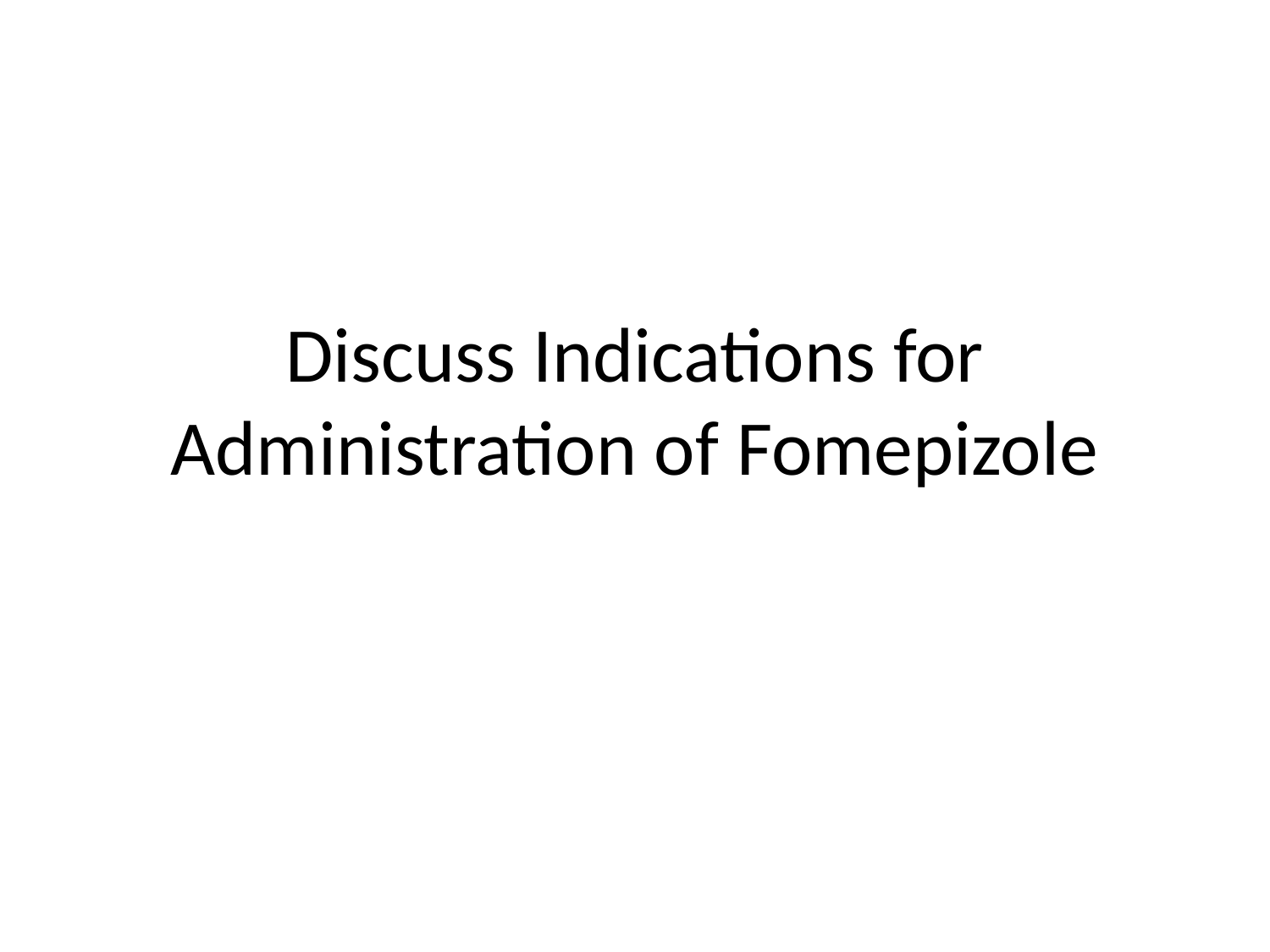

# Discuss Indications for Administration of Fomepizole

## Slide 24
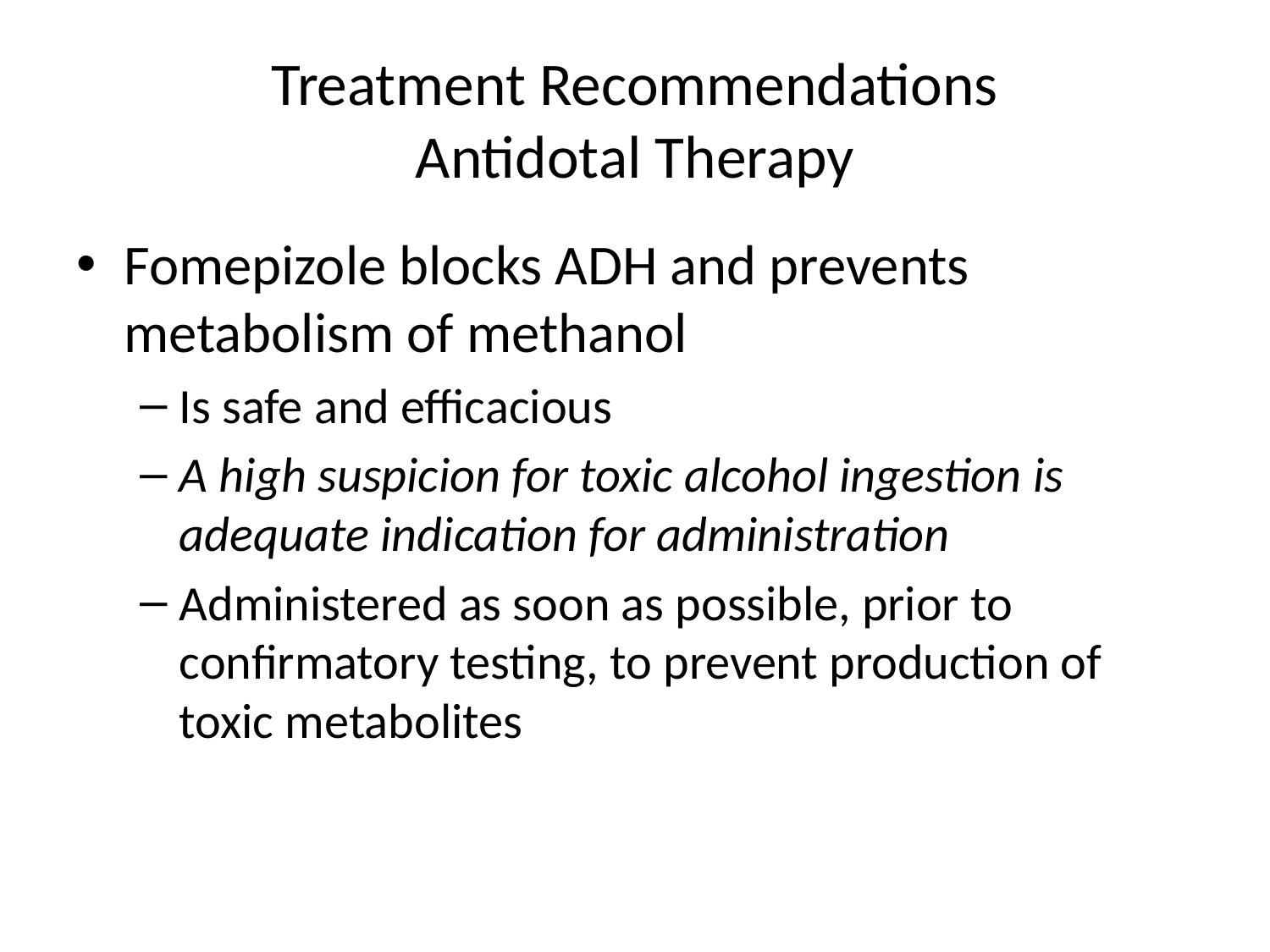

# Treatment Recommendations Antidotal Therapy
Fomepizole blocks ADH and prevents metabolism of methanol
Is safe and efficacious
A high suspicion for toxic alcohol ingestion is adequate indication for administration
Administered as soon as possible, prior to confirmatory testing, to prevent production of toxic metabolites

## Slide 25
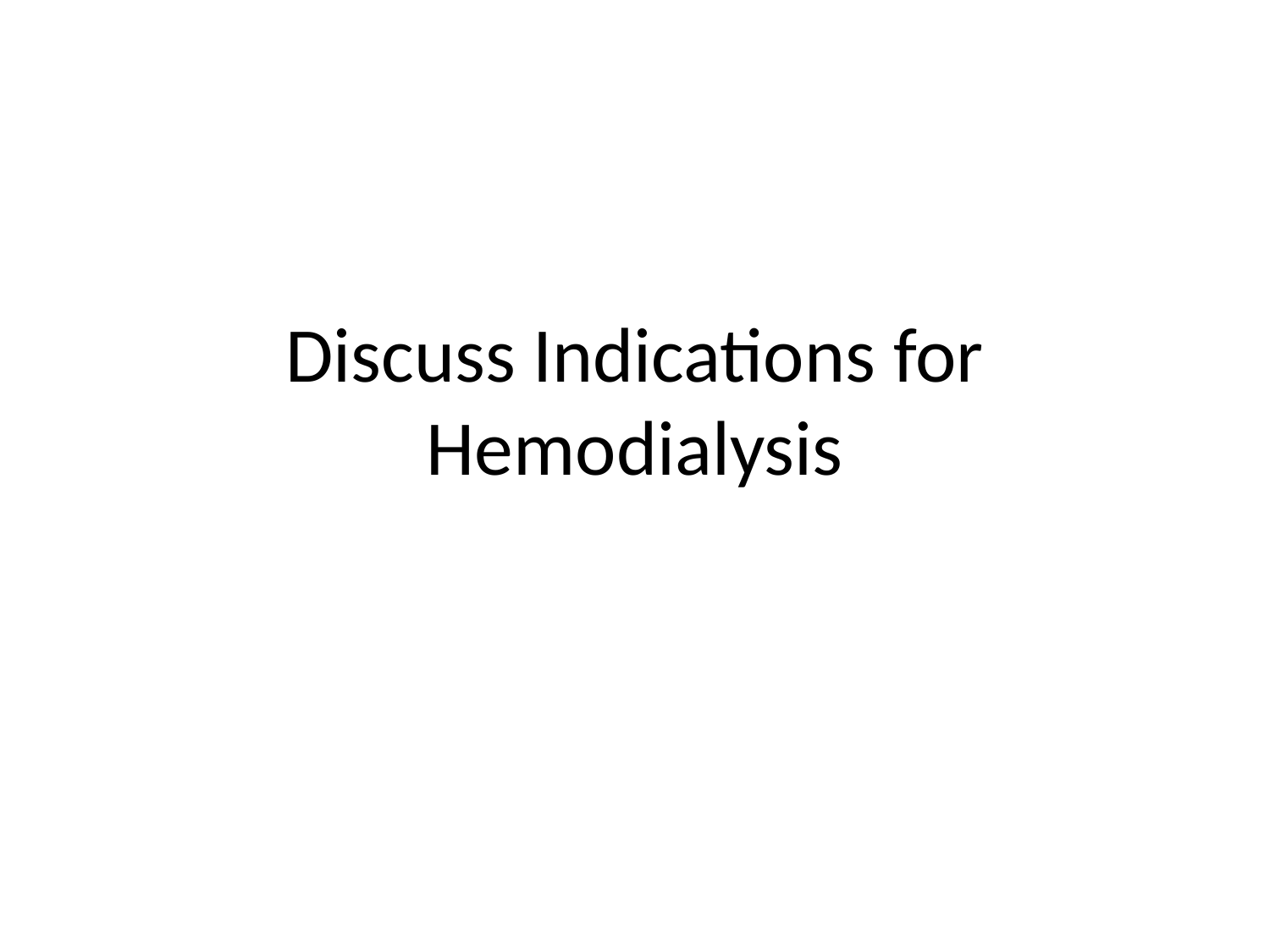

# Discuss Indications for Hemodialysis

## Slide 26
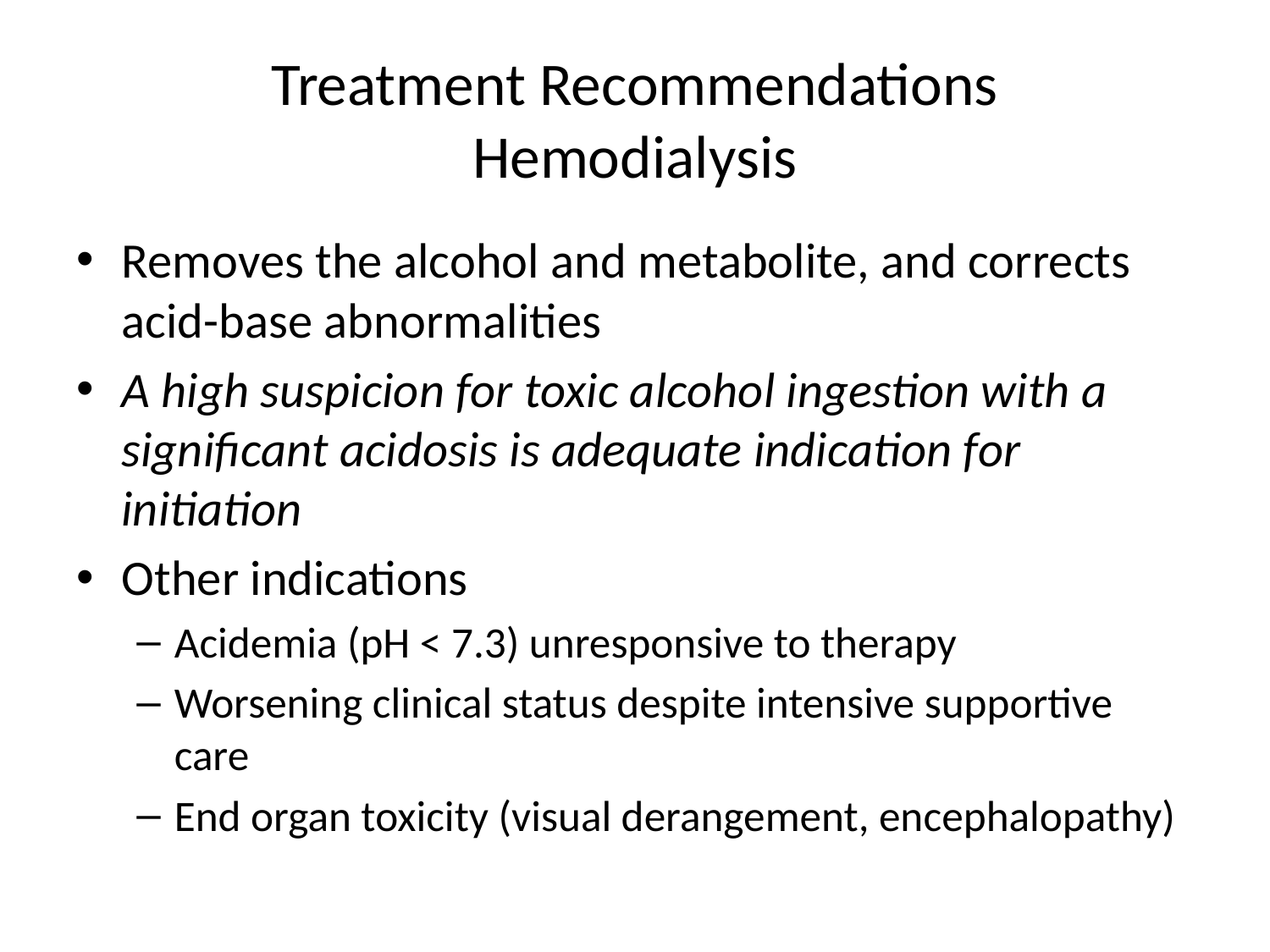

# Treatment Recommendations Hemodialysis
Removes the alcohol and metabolite, and corrects acid-base abnormalities
A high suspicion for toxic alcohol ingestion with a significant acidosis is adequate indication for initiation
Other indications
Acidemia (pH < 7.3) unresponsive to therapy
Worsening clinical status despite intensive supportive care
End organ toxicity (visual derangement, encephalopathy)

## Slide 27
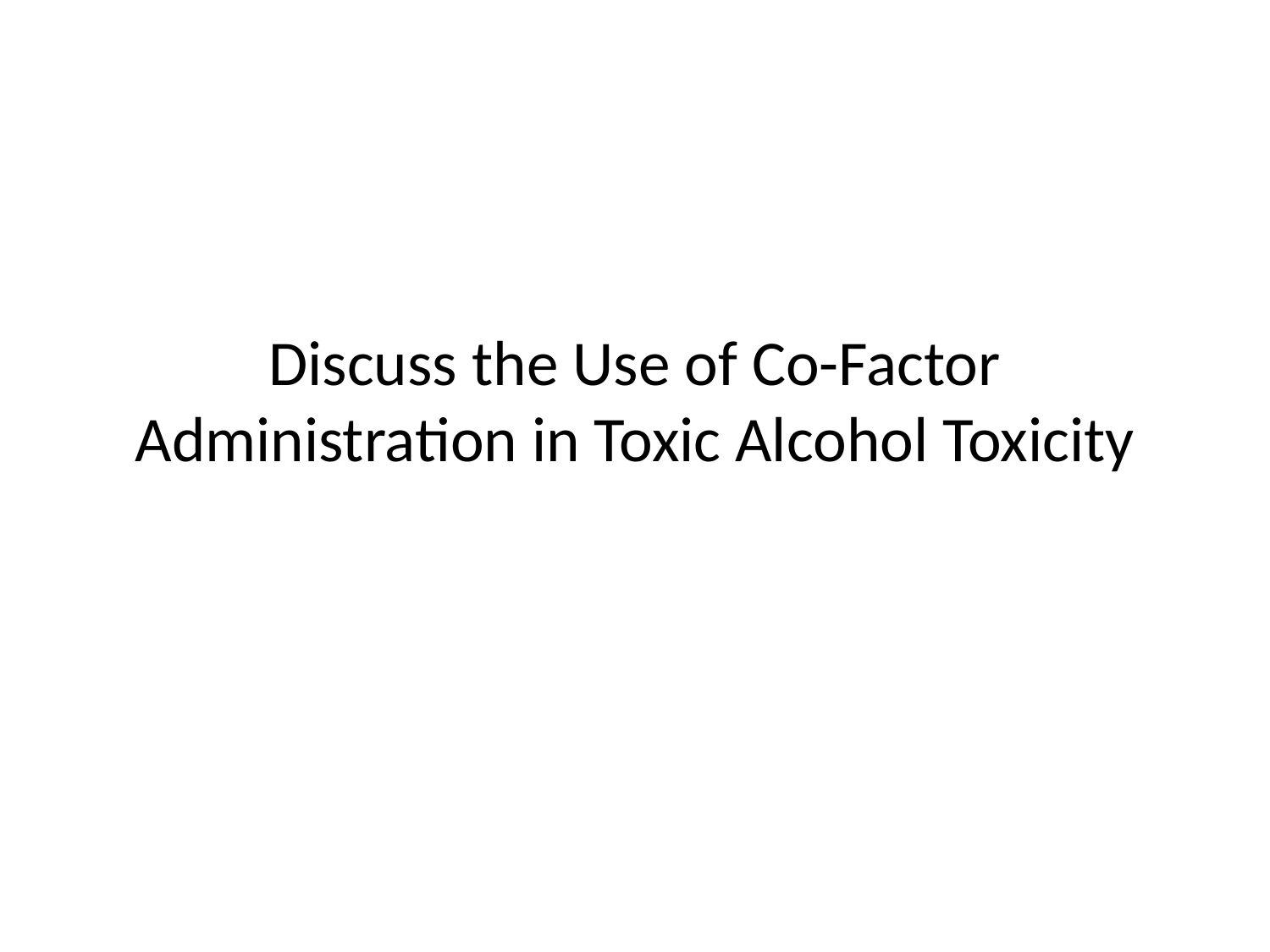

# Discuss the Use of Co-Factor Administration in Toxic Alcohol Toxicity

## Slide 28
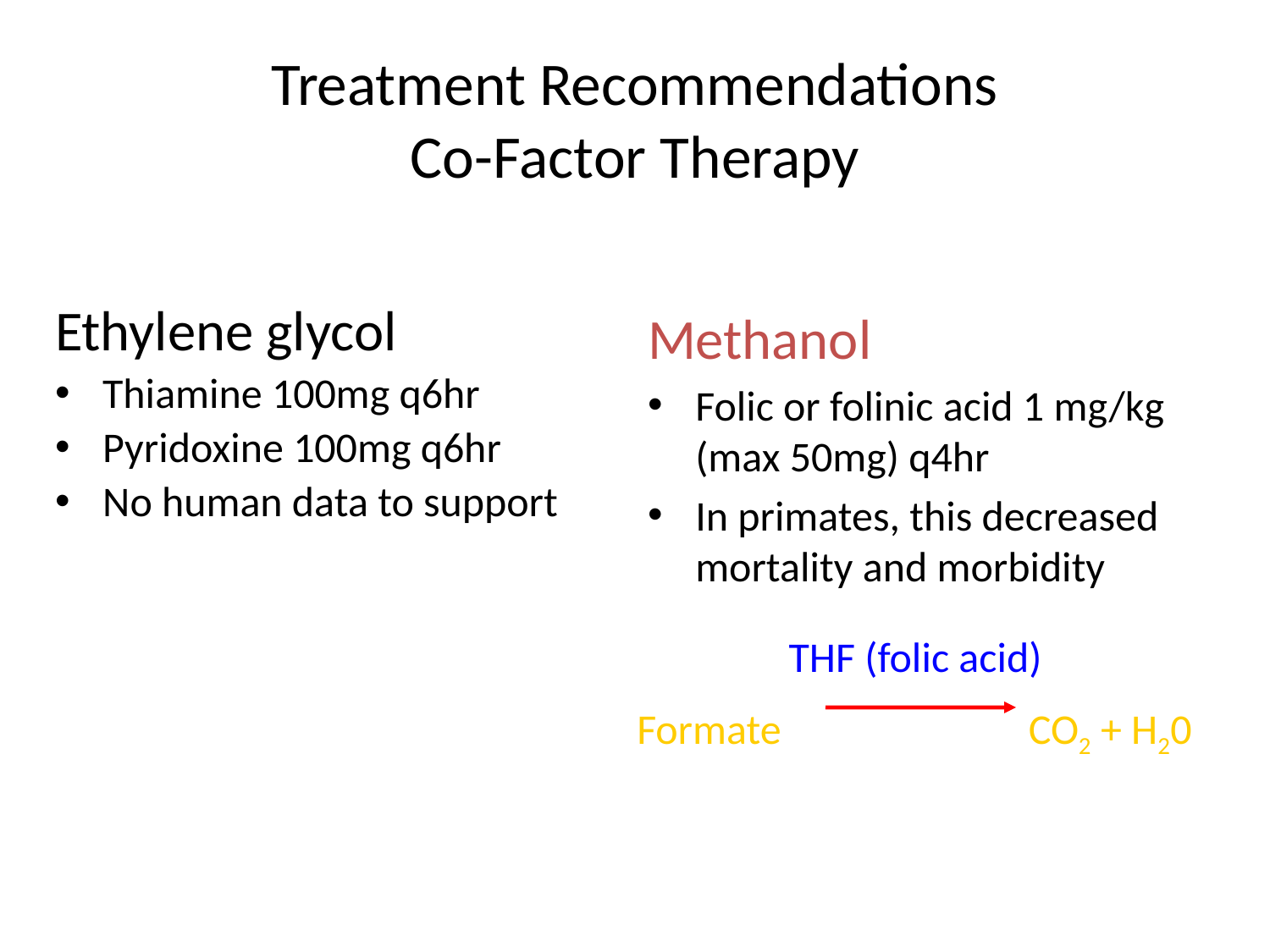

# Treatment Recommendations Co-Factor Therapy
Ethylene glycol
Thiamine 100mg q6hr
Pyridoxine 100mg q6hr
No human data to support
Methanol
Folic or folinic acid 1 mg/kg (max 50mg) q4hr
In primates, this decreased mortality and morbidity
THF (folic acid)
Formate
CO2 + H20

## Slide 29
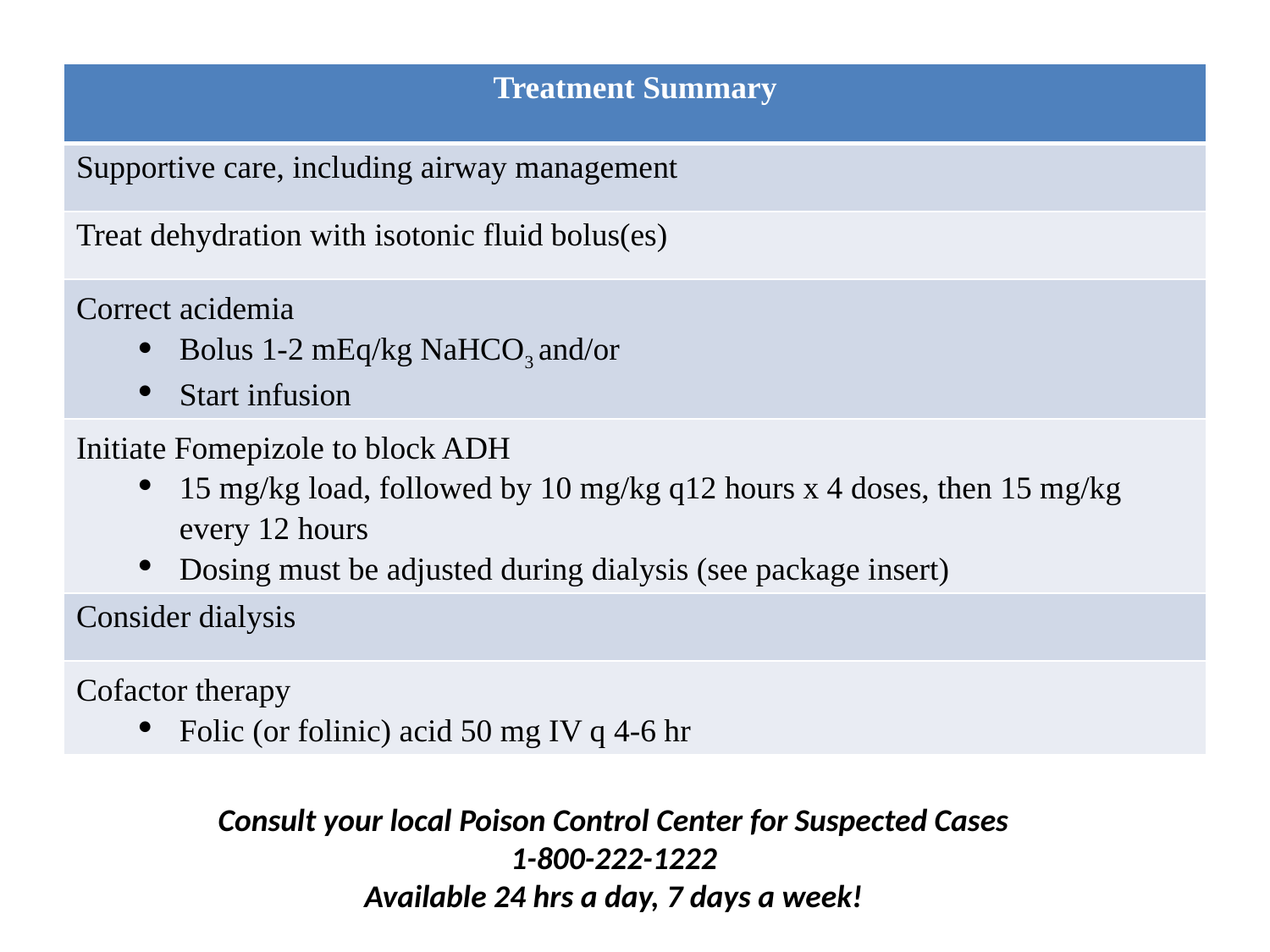

| Treatment Summary |
| --- |
| Supportive care, including airway management |
| Treat dehydration with isotonic fluid bolus(es) |
| Correct acidemia Bolus 1-2 mEq/kg NaHCO3 and/or Start infusion |
| Initiate Fomepizole to block ADH 15 mg/kg load, followed by 10 mg/kg q12 hours x 4 doses, then 15 mg/kg every 12 hours Dosing must be adjusted during dialysis (see package insert) |
| Consider dialysis |
| Cofactor therapy Folic (or folinic) acid 50 mg IV q 4-6 hr |
Consult your local Poison Control Center for Suspected Cases
1-800-222-1222
Available 24 hrs a day, 7 days a week!

## Slide 30
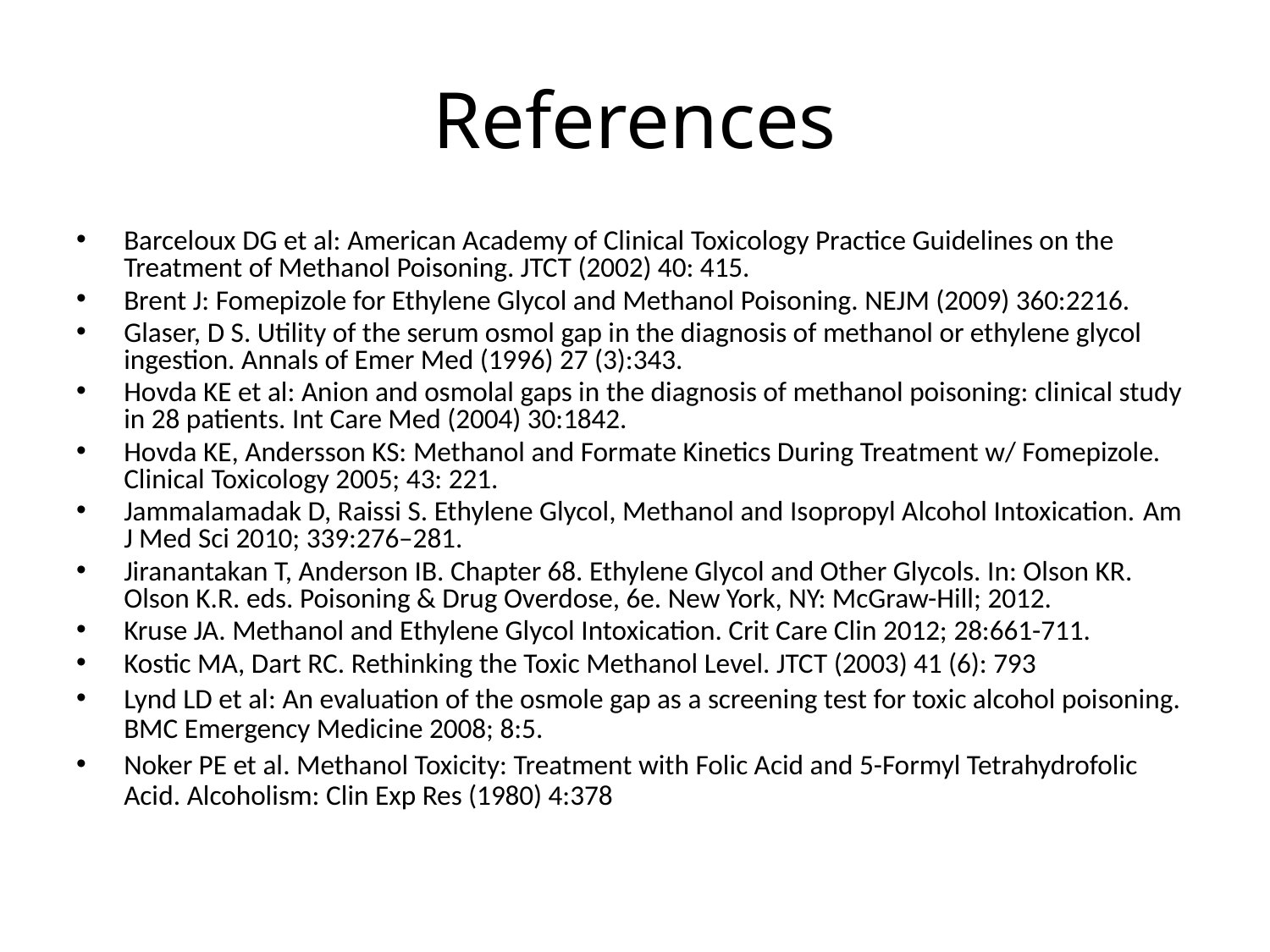

# References
Barceloux DG et al: American Academy of Clinical Toxicology Practice Guidelines on the Treatment of Methanol Poisoning. JTCT (2002) 40: 415.
Brent J: Fomepizole for Ethylene Glycol and Methanol Poisoning. NEJM (2009) 360:2216.
Glaser, D S. Utility of the serum osmol gap in the diagnosis of methanol or ethylene glycol ingestion. Annals of Emer Med (1996) 27 (3):343.
Hovda KE et al: Anion and osmolal gaps in the diagnosis of methanol poisoning: clinical study in 28 patients. Int Care Med (2004) 30:1842.
Hovda KE, Andersson KS: Methanol and Formate Kinetics During Treatment w/ Fomepizole. Clinical Toxicology 2005; 43: 221.
Jammalamadak D, Raissi S. Ethylene Glycol, Methanol and Isopropyl Alcohol Intoxication. Am J Med Sci 2010; 339:276–281.
Jiranantakan T, Anderson IB. Chapter 68. Ethylene Glycol and Other Glycols. In: Olson KR. Olson K.R. eds. Poisoning & Drug Overdose, 6e. New York, NY: McGraw-Hill; 2012.
Kruse JA. Methanol and Ethylene Glycol Intoxication. Crit Care Clin 2012; 28:661-711.
Kostic MA, Dart RC. Rethinking the Toxic Methanol Level. JTCT (2003) 41 (6): 793
Lynd LD et al: An evaluation of the osmole gap as a screening test for toxic alcohol poisoning. BMC Emergency Medicine 2008; 8:5.
Noker PE et al. Methanol Toxicity: Treatment with Folic Acid and 5-Formyl Tetrahydrofolic Acid. Alcoholism: Clin Exp Res (1980) 4:378
