# Supplementary material for: Toxic Alcohol Ingestion/Methanol Ingestion
Source: MedEdPORTAL. 2018 Aug 17;14:10740. doi: 10.15766/mep_2374-8265.10740 (PMC6342378; doi:10.15766/mep_2374-8265.10740)
Supplement: Supplementary file 1 — A. Methanol Simulation Case.docx B. Methanol Supplemental Case Findings.ppt C. Methanol Questionnaire.docx D. Methanol Evaluation Form.doc E. Methanol Case Debriefing.pptx [file mep-14-10740-s001.zip › D._Methanol_Evaluation_Form.doc]

Participant’s name:

# Methanol Toxicity

# Evaluation Novice Apprentice Practitioner

Communication present between team members

Appropriate division of tasks

Case Evaluation

Check blood sugar on altered mental status patient

Performed a focused history and physical exam based on complaint

Resuscitation started

Notes:

Intubate patient

Appropriate development of differential diagnosis based on AGMA

Start fomepizole

Acknowledging need for Nephrology consultation for dialysis

Appropriate disposition to ICU
